# Type I collagen and fibromodulin enhance the tenogenic phenotype of hASCs and their potential for tendon regeneration

**DOI:** 10.1038/s41536-023-00341-z

**Published:** 2023-12-14

**Authors:** Tian Tu, Yuan Shi, Boya Zhou, Xiaoyu Wang, Wenjie Zhang, Guangdong Zhou, Xiumei Mo, Wenbo Wang, Jinglei Wu, Wei Liu

**Affiliations:** 1 Department of Plastic and Reconstructive Surgery, Shanghai Ninth People’s Hospital, Shanghai Jiao Tong University School of Medicine, Shanghai, 200011 China; 2 Plastic and Aesthetic Center, The First Affiliated Hospital, Zhejiang University School of Medicine, Hangzhou, Zhejiang Province 310003 China; 3 Department of Burn and Plastic Surgery, First Affiliated Hospital of Soochow University, Suzhou, Jiangsu Province 215000 China; 4 Shanghai Engineering Research Center of Nano-Biomaterials and Regenerative Medicine, College of Biological Science and Medical Engineering, Donghua University, Shanghai, 201620 P. R. China; 5 National Tissue Engineering Center of China, Shanghai, 200241 China

**Keywords:** Mesenchymal stem cells, Stem-cell research

## Abstract

Our previous work demonstrated the tendon-derived extracellular matrix (ECM) extracts as vital niches to specifically direct mesenchymal stem cells towards tenogenic differentiation. This study aims to further define the effective ECM molecules capable of teno-lineage induction on human adipose-derived stem cells (hASCs) and test their function for tendon engineering. By detecting the teno-markers expression levels in hASCs exposed to various substrate coatings, collagen I (COL1) and fibromodulin (FMOD) were identified to be the key molecules as a combination and further employed to the modification of poly(L-lactide-*co*-ε-caprolactone) electrospun nanoyarns, which showed advantages in inducting seeded hASCs for teno-lineage specific differentiation. Under dynamic mechanical loading, modified scaffold seeded with hASCs formed neo-tendon in vitro at the histological level and formed better tendon tissue in vivo with mature histology and enhanced mechanical properties. Primary mechanistic investigation with RNA sequencing demonstrated that the inductive mechanism of these two molecules for hASCs tenogenic differentiation was directly correlated with positive regulation of peptidase activity, regulation of cell-substrate adhesion and regulation of cytoskeletal organization. These biological processes were potentially affected by LOC101929398/has-miR-197-3p/TENM4 ceRNA regulation axis. In summary, COL1 and FMOD in combination are the major bioactive molecules in tendon ECM for likely directing tenogenic phenotype of hASCs and certainly valuable for hASCs-based tendon engineering.

## Introduction

Tendon extracellular matrices (ECM), especially fibromodulin (Fmod) and biglycan (BGN), constitute the vital ECM niche for in vivo resided tendon stem/progenitor cells and properly prevent their shifting from tenogenic lineage to osteogenic/chondrogenic lineages^1^. Therefore, the role of tendon ECM in tendon regeneration has been extensively investigated using decellularized tendon scaffold or tendon ECM extract^2–5^. In most of these studies, total ECM extracts were usually utilized as the natural scaffold directly or used for artificial scaffold modification^6,7^. Tendon ECM modified electrospun scaffold has been proven to effectively induce tenogenic differentiation of mesenchymal stem cells as previously reported^7^. Although considerable progress has been achieved in this field, the actual tendon ECM components with teno-inductive effects to enhance the tenogenic phenotype and functions of seeded stem cells under tissue engineering application conditions remain largely unknown, and thus functional tendon engineering is less likely to be improved^8^.

Previous studies have suggested that several components extracted from tendon ECM play a critical role in inducing the tenogenic phenotype^4,9^. Among these components, type I collagen (COL1) makes up the bulk of tendon ECM, so it has been most frequently used as a fundamental bioactive interface in the context of tissue engineering to provide more biocompatible adhesion points and bioactive signals for the cells seeded on the modified scaffolds^10,11^. However, since collagens are generally the infrastructures of ECM structures in various tissues and apparently lack the ability to exert specific tenogenic induction effect, it is necessary to complement them with other teno-potent inducers. Besides, other non-collagenous components, mainly comprised of small leucine-rich proteoglycans (SLRPs) such as Fmod, BGN and decorin (DCN), have also been demonstrated to exert biological effect on enhancing tenogenic differentiation, assisting matrix organization and preventing ectopic chondrogenesis/osteogenesis during tendon development^1,8,12^. Impaired tendon development in juveniles or chronic tendinopathy in adults, usually manifested as ectopic ossification, can be observed in these SLRPs-knockdown animal models, suggesting a deviation in the tenogenic phenotype of the native tendon stem/progenitor cells^1,12^. It has been shown that several tendon-derived SLRP molecules (e.g., Fmod, BGN, and DCN) provide stronger specific tendon-inducible signals in a collagen-rich environment^13–15^. Since the tendon stem/progenitor cells are also members of the mesenchymal stem cells (MSCs) family, combining COL1 and SLRPs proteins might be the promising options for tenogenic induction of MSCs from diverse tissue sources^16^.

In addition to appropriate biochemical signals provided by ECM molecules, the non-biological performance of basic scaffold for carrying ECM components is also crucial amid the cell-scaffold construction^17^. In our previous work, a novel electrospun nanoyarn network that was morphologically and structurally similar to that of the native tendon ECM was prepared by blending poly(L-lactide-*co*-ε-caprolactone) (PLCL) and COL1^10,18^. Since collagen in the nanoyarns could provide abundant amino groups, the SLRPs could be easily crosslinked to blended yarns by 1-ethyl-3-(3-dimethylaminopropyl)carbodiimide (EDC)/N-hydroxysuccinimide (NHS) reaction without introducing redundant carbon chain^19^. Based on these works, we hypothesize that the group of ECM molecules composed of COL1 and various SLRPs may serve as the important niche biofactors after been immobilized on nanoyarn scaffolds, and then render them teno-potent for inducing tenogenic differentiation of seeded stem cells in vitro and tendon regeneration in vivo.

In present study, the human adipose-derived stem cells (hASCs) were employed as a research object for proof-of-concept study because of their safe access and potential for application in homologous allogeneic tendon regeneration^20^. To validate the hypothesis experimentally, the most effective teno-potent ECM molecules were screened and evaluated by direct interaction with hASCs for tenogenic induction. The most potent ECM molecules were then used to modify the electrospun PLCL nanoyarns scaffold for various experiments in vitro and tendon engineering in vivo. In addition, RNA sequencing was employed to explore the potential mechanism by which teno-potent ECMs regulate the hASCs tenogenic differentiation and tendon regeneration.

## Results

### Schematic of experiment design

**Fig. 1:**
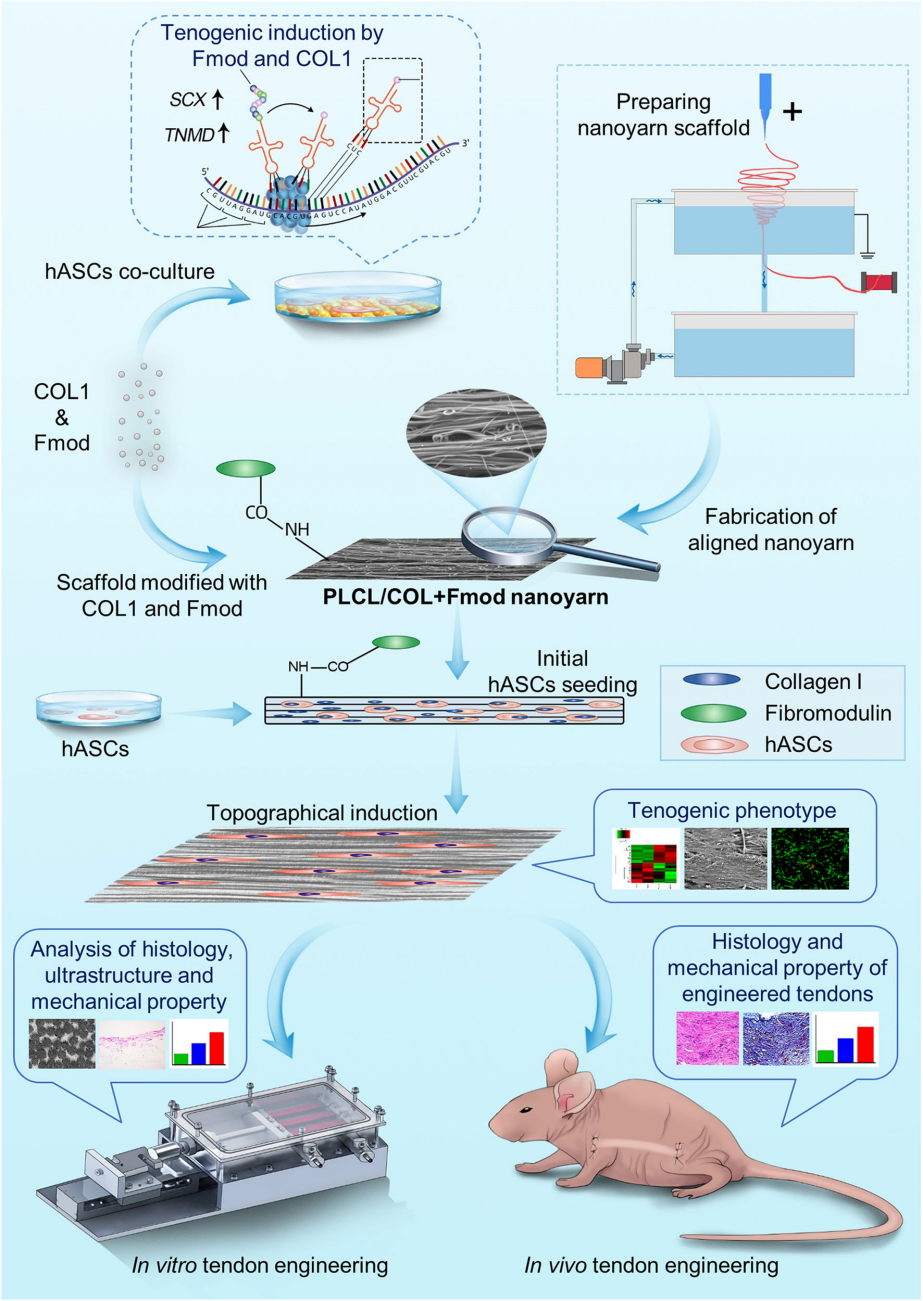
The schematic illustration of the experimental design.

As illustrated in Fig. 1, COL1 and Fmod were used to modify PLCL nanoyarn scaffolds to generate PLCL/COL1+Fmod scaffolds, since hASCs expressed higher levels of tenogenic genes on petri-dish coated with COL1 and Fmod. The nanoyarn scaffolds seeded with hASCs were cultured under general condition for inducing tenogenic phenotype. Cytocompatibility and alteration at the transcriptomic level were examined at the 7th day after cell seeding. Subsequently, the cell-scaffold construct was cultivated in vitro in a customized bioreactor at both static and mechanical loading conditions, or implanted subcutaneously in nude mouse to form neo-tendon tissues in vivo after 7 days of in vitro culture.

### Enhanced tenogenic gene expression of hASCs on the surface coated with COL1 and Fmod

**Fig. 2:**
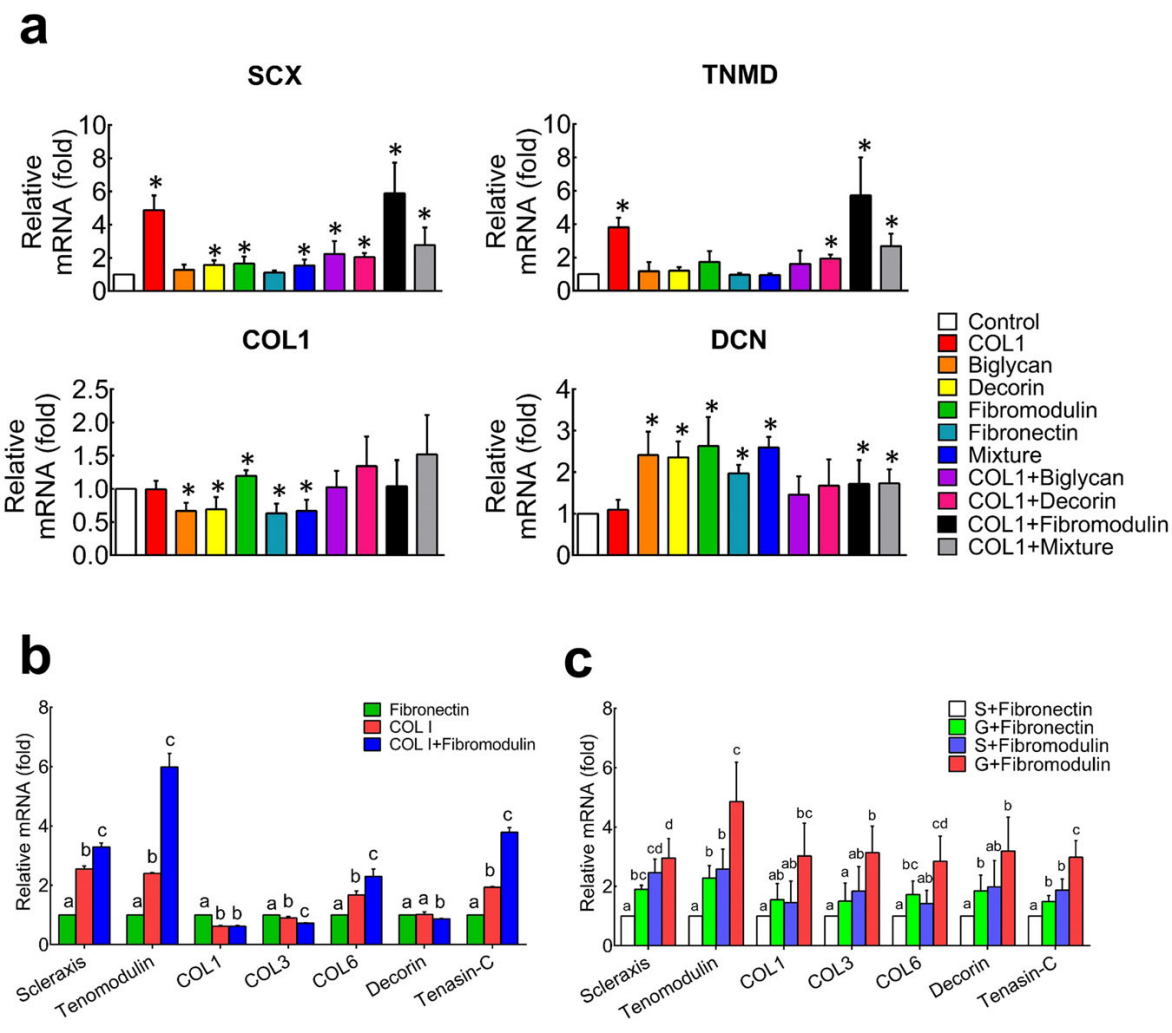
The hASCs expressed higher levels of tenogenic genes on substrates coated with collagen I and fibromodulin. **a** Screening of teno-inductive ECM components via gene expression of tenogenic markers of *SCX*, *TNMD*, *COL1* and *DCN*. *p < 0.05 comparing to control. **b** hASCs expressed higher levels of tenogenic genes on smooth polydimethylsiloxane membranes coated with both collagen I and fibromodulin using fibronectin coating as a control. **c** Fibromodulin coated microgroove substrate promoted tenogenic differentiation of hASCs synergistically. Significant differences existed between two groups labeled with different letters. SCX scleraxis, TNMD tenomodulin, Col1 collagen I, DNC decorin, S smooth surface, G microgroove surface. All error bars indicate standard deviation.

To identify the effective ECM molecules that are capable of providing a reliable tenogenic niche for hASCs, five candidate components of tendon ECM, including COL1, BGN, DCN, Fmod and fibronectin (FN), were coated on the culture dish alone or in various combinations. As shown in Fig. 2a, the fold change levels of *scleraxis* (*SCX*) and *tenomodulin* (*TNMD*) in COL1 group and COL1+Fmod group were significantly higher than those of other groups (*p* < 0.05). The hASCs cultured with non-collagenous components expressed high levels of *DCN* (*p* < 0.05), whereas the expression levels of *SCX*, *TNMD* and *COL1* were not well enhanced in these groups. On the other hand, the expression level of osteogenic and adipogenic lineage markers, such as *Runt-related transcription factor 2* (*RUNX2*), *Osteocalcin* (*OCN*), *CCAAT enhancer binding protein alpha* (*C/EBPα*), *Adiponectin* and *peroxisome proliferator-activated receptor gamma* (*PPAR-γ*), did not increase, except for alkaline phosphatase (*ALPL*), which significantly increased upon dual induction (Supplementary Fig. 1). Taken together, the screen test results suggested that the COL1 or COL1 plus Fmod may serve as the candidate components for the tenogenic induction of hASCs.

To further test the induction effect of ECM molecules on surfaces with different topography, the tenogenic differentiation of hASCs was observed on (polydimethylsiloxane) PDMS membrane, which has reduced stiffness than Petri dish and can be tailored with parallel microgrooved surface. As revealed in Fig. 2b, expression levels of *SCX*, *TNMD*, *Collagen VI* (*COL6*) and *Tenascin-C* were significantly enhanced in COL1 and Fmod groups when hASCs were cultured on the flat PDMS (*p* < 0.05). Moreover, the highest expression levels of these genes in COL1 + Fmod group proved the synergetic effect between COL1 and Fmod (Fig. 2b, c). On the other hand, the induction effect of Fmod could be enhanced on the aligned microgrooved surface, as the expression levels of *SCX*, *TNMD*, *Collagen III* and *Tenascin-C* were significantly increased compared with the flat FN group and the microgrooved FN group (Fig. 2c, *p* < 0.05).

### Preparation and characterization of nanoyarn scaffold

**Fig. 3:**
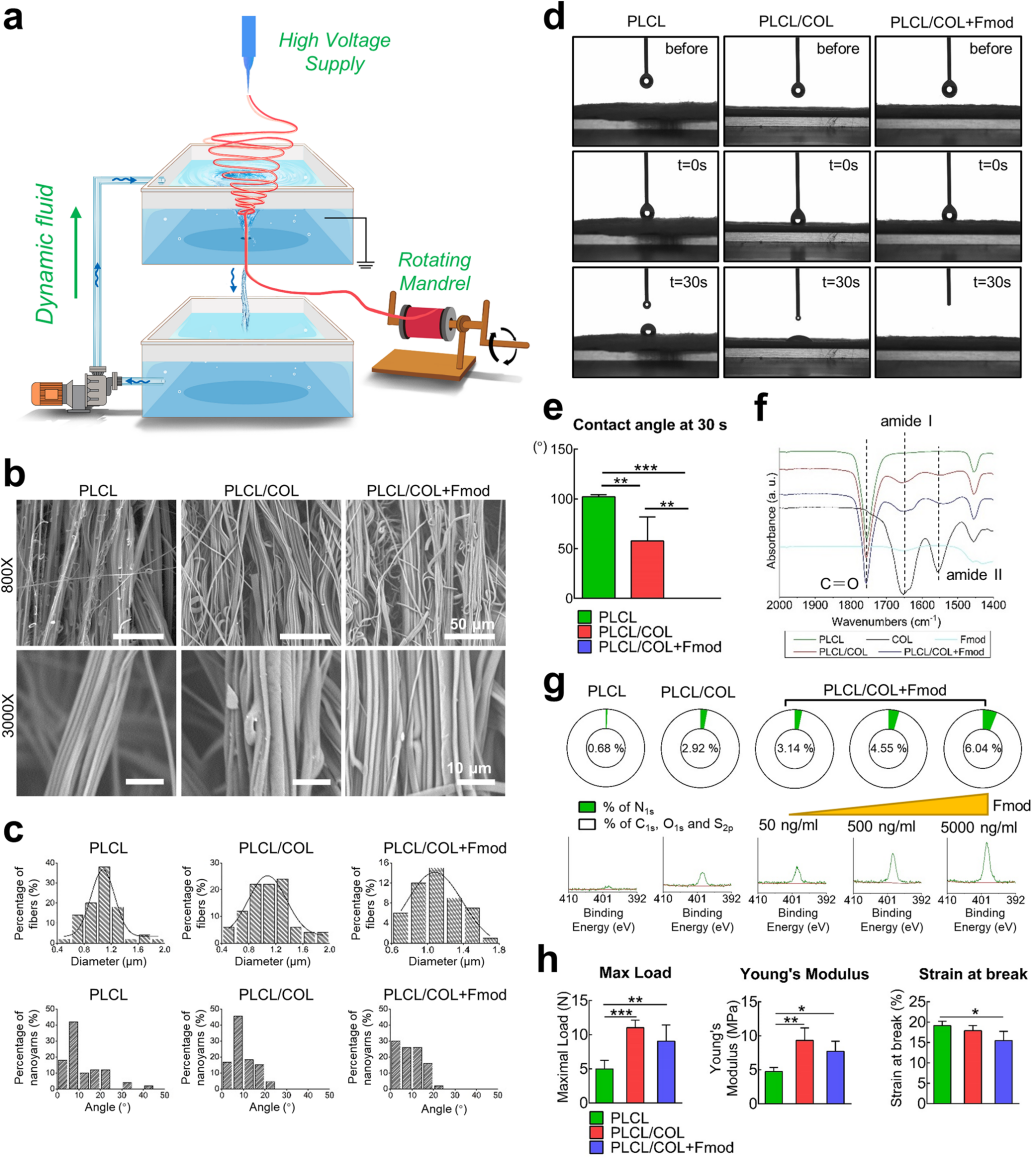
Fabrication and characterization of electrospun nanoyarn scaffold. **a** Illustrated working principle for generating electrospun nanoyarns; **b** SEM images of nanoyarn scaffold with similar surface morphology among different groups; **c** Frequency distribution chart of individual fiber diameters and the angles between adjacent yarns; **d** Processes of water droplet spreading on scaffold surface recorded before, 0 s and 30 s after water-material contact; **e** Quantitative measurement of water contact angles at 30 s; **f** FTIR spectra of different scaffolds, and COL1 and Fmod revealed overlapped characteristic absorption peaks of C = O bond, amid I group and amid II group; **g** XPS assay to show the dose-dependent increasing proportion of nitrogen on the surface of nanoyarns; **h** Histogram illustration of mechanical properties of different nanoyarn scaffolds. All error bars indicate standard deviation. *p < 0.05, **p < 0.01, ***p < 0.001.

The working principle of fabrication system was illustrated in Fig. 3a. SEM images showed that the fiber bundles in PLCL scaffold, PLCL/COL scaffold, and PLCL/COL+Fmod scaffold were basically aligned in principle with the angles mainly ranging from 0° to 20° (Fig. 3b, c). Higher-magnification images revealed that these nanofibers within one yarn were regionally aligned and closely attached with each other. Quantitatively, the average diameters of individual fibers were about 1.1 ± 0.3 μm for each group (Fig. 3b, c). No significant difference in yarn diameters was detected (*p* > 0.05).

Water droplets spread faster on the scaffolds modified with COL1 and Fmod. As recorded in Fig. 3d, the water droplets spread completely on the PLCL/COL + Fmod scaffold and partially spreads on the PLCL/COL scaffold at 30 s, while the droplets remained nearly spherical on the PLCL scaffold at the same time point. The measurement data showed the contact angle at 30 s of PLCL, PLCL/COL and PLCL/COL+Fmod scaffold were 102 ± 2°, 58 ± 24° and 0 ± 0°, respectively, indicating the best hydrophilicity of these nanoyarns when modified with COL1 and Fmod (Fig. 3e, *p* < 0.05).

The chemical composition of the modified nanoyarns was examined by ATR-FTIR and XPS. The ATR-FTIR analysis revealed the characteristic peak of C = O bond at 1755 cm^−1^, which was shared by all three types of scaffolds. The modified scaffolds (PLCL/COL and PLCL/COL+Fmod scaffold) shared the characteristic peaks of amide I and II group with COL1 at 1650 cm^−1^ and 1550 cm^−1^, respectively (Fig. 3f). However, the absorption peak of amide II was not so obvious as that of COL1 as illustrated in Fig. 3f. Since nitrogen was widely considered as a symbolic element of proteins when compared to aliphatic polyester, the XPS analysis showed the proportion variation of nitrogen on the surface of PLCL, PLCL/COL and PLCL/COL+Fmod scaffolds, which was revealed by the amplitude of the binding energy peak at 398 eV. As shown in Fig. 3g, the nitrogen content in PLCL/COL scaffolds was much higher than that in PLCL scaffolds. After further modification with Fmod, the nitrogen content in the PLCL/COL+Fmod scaffolds were obviously higher than those in the PLCL/COL scaffolds, and increased in a dose-dependent manner as the concentration of Fmod was elevated from 50 ng/mL to 5000 ng/mL. In contrast, the proportion of other elements, including C, O, and S, were reduced on the surface of PLCL/COL+Fmod scaffolds in a dose-dependent manner, suggesting that nitrogen was introduced into the scaffolds after crosslinking with Fmod.

Changes in mechanical properties revealed higher load bearing capacity and stiffness but lower malleability after modification. As revealed in Fig. 3h and Supplementary Fig. 2, the maximal load of the PLCL, PLCL/COL, and PLCL/COL+Fmod scaffolds was 5.0 ± 1.3 N, 11.0 ± 1.1 N and 9.1 ± 2.4 N, respectively (*p* < 0.05). The Young’s modulus of the PLCL, PLCL/COL, and PLCL/COL+Fmod scaffolds was 4.7 ± 0.6 MPa, 9.3 ± 1.8 MPa and 7.7 ± 1.5 MPa, respectively (*p* < 0.05). The strain at break of the PLCL, PLCL/COL, and PLCL/COL+Fmod scaffolds was 19.1 ± 1.1%, 17.9 ± 1.3% and 15.5 ± 2.3%, respectively (*p* < 0.05).

### Phenotypic identification of hASCs

The flow cytometry assay showed that these adipose-derived cells expressed characteristic surface markers of mesenchymal stem cells including CD13, CD44, CD73, CD90 and CD105. Meanwhile, the negative markers of mesenchymal stem cells including CD31, CD45, and HLA-DR were not detected (Supplementary Fig. 3).

### Elongated cellular morphology and enhanced collagen deposition of hASCs on dual-modified yarns

**Fig. 4:**
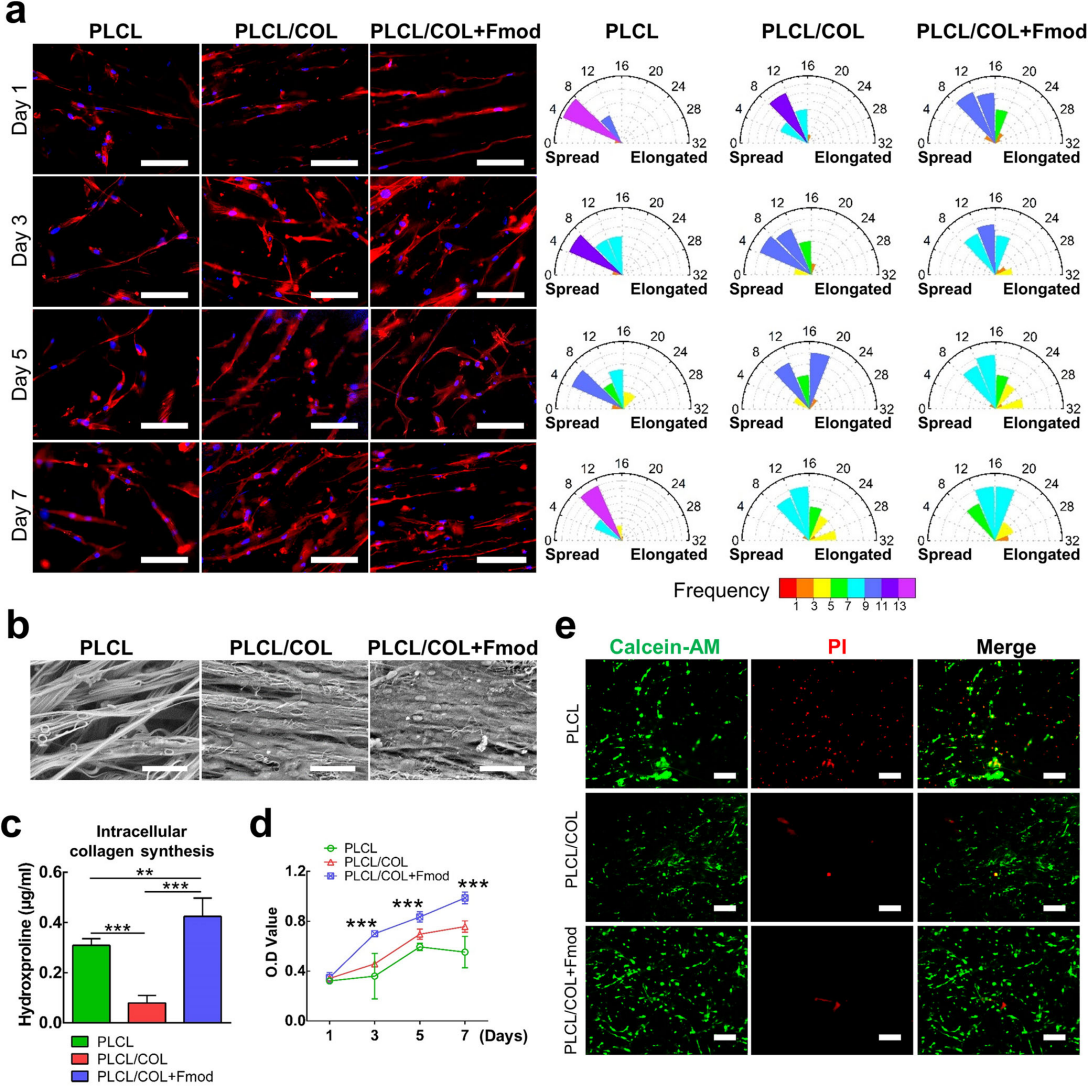
Modification with COL1 and Fmod significantly improved the in vitro cytocompatibility of nanoyarns. **a** Cytoskeleton staining revealed the accelerated spreading process of hASCs along the PLCL/COL+Fmod nanoyarns. The highest cell aspect ratio was also observed in PLCL/COL+Fmod group. Magnification, 200×; Bar 100 μm. **b** SEM images visually presented the most abundant matrix deposition on PLCL/COL+Fmod scaffold. Magnification, 800×; Bar = 50 μm. **c** Quantitation of intracellular hydroxyproline indicated alteration of biosynthesis level of collagen. **d** Cell proliferation curve was generated by CCK-8 assay. **e** Live/dead staining revealed enhanced cell viability on two types of modified scaffolds. All error bars indicate standard deviation. **p < 0.01, ***p < 0.001.

As revealed in Fig. 4a, hASCs stably adhered to the yarns and gradually exhibit their elongated morphology after 7 days of culture on three types of nanoyarn scaffolds. However, the hASCs on the modified nanoyarn scaffold showed higher cell aspect ratios. The averaged cell aspect ratios of the cells on PLCL, PLCL/COL, and PLCL/COL + Fmod scaffolds were respectively 7.1 ± 2.3, 10.5 ± 3.4, 13.7 ± 4.6 on the 1st day (*p* < 0.05), 8.8 ± 3.2, 9.1 ± 4.1, 16.9 ± 6.2 on the 3rd day (*p* < 0.05), 11.0 ± 5.3, 13.8 ± 4.6, 17.0 ± 7.6 on the 5th day (*p* < 0.05), and 9.2 ± 3.1, 11.7 ± 6.1, 17.2 ± 6.2 on the 7th day (*p* < 0.05). The frequency distribution diagram also revealed the highest cell aspect ratio of hASCs on PLCL/COL+Fmod scaffolds (Fig. 4a).

The SEM images showed enhanced collagen deposition of hASCs on the 7th day of cell culture on modified scaffolds (Fig. 4b). In detail, little extracellular matrix deposition was observed around the cells on PLCL scaffold where the outline of cell morphology was clearly visible. On the contrary, collagen sheet formed on PLCL/COL scaffold and the outline of cell morphology became ambiguous when compared to those in PLCL group. A thick collagen layer was observed on PLCL/COL+Fmod scaffolds where cells were already embedded in the collagen layer. Nonetheless, the intracellular collagen synthesis, which was detected by hydroxyproline quantification assay, showed a different pattern from the results visually observed in SEM images. Collagen synthesis decreased in the PLCL/COL group but increased significantly in the PLCL/COL+Fmod group when compared to that in the PLCL group (Fig. 4c, *p* < 0.05).

### Cell proliferation and viability on yarns

The hASCs proliferated at the fastest rate in the PLCL/COL+Fmod group and at a slower rate in the PLCL/COL group as shown by CCK-8 assay, in which significant differences were detected on days 3, 5, and 7 (Fig. 4d, *p* < 0.05). Live/dead double staining also revealed fewer dead cells on the modified yarn scaffold (Fig. 4e).

### COL1 and Fmod accelerated tissue maturation of hASCs-yarns constructs in vitro

**Fig. 5:**
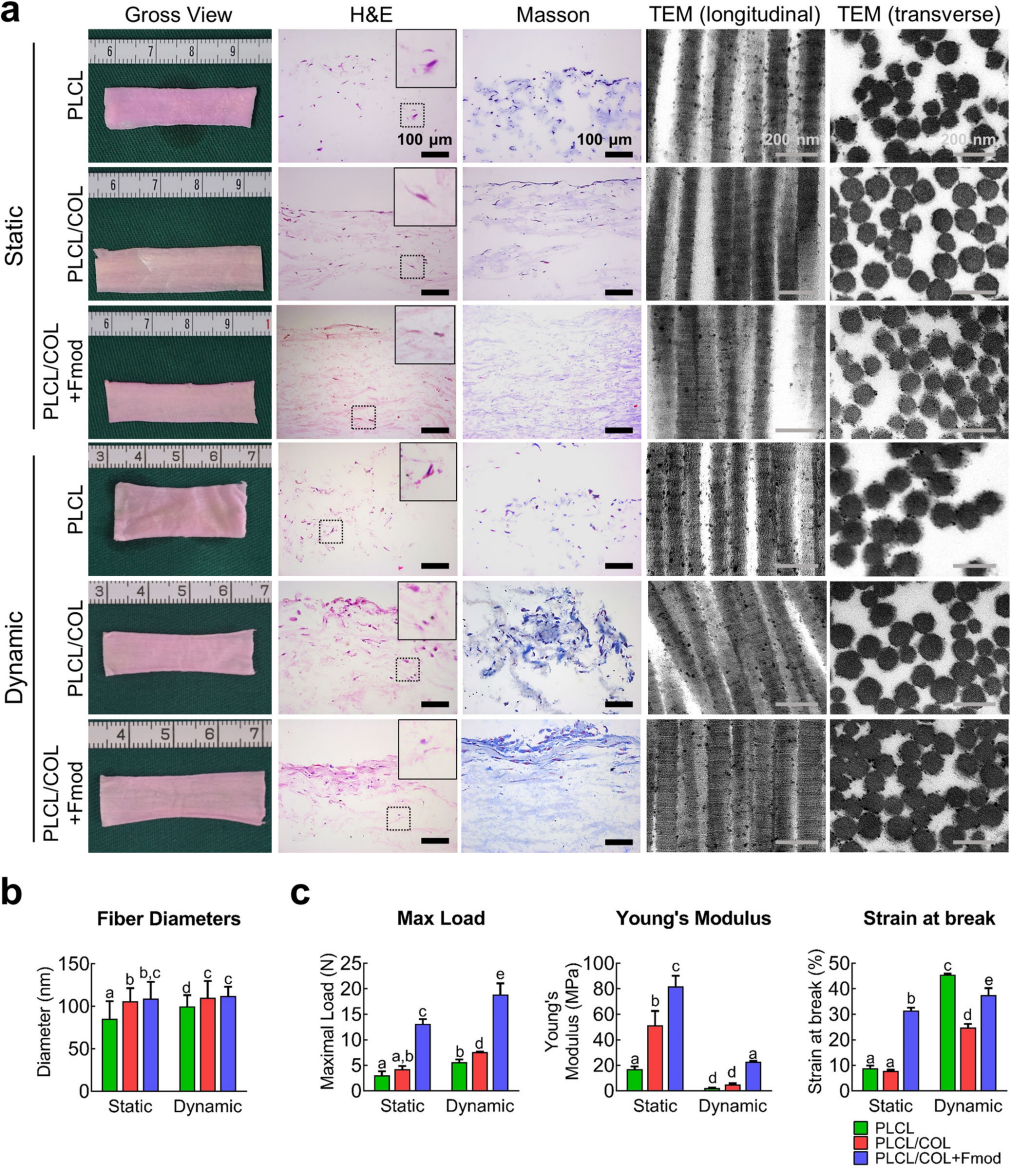
COL1 combined with Fmod promoted the in vitro neo-tendon formation under dynamic mechanical stimulation. **a** The gross view, H&E staining, Masson trichrome staining and TEM images were observed after the 4-week culture of cell-scaffold complex under static or dynamic condition. The contents of the dotted box were enlarged and presented in the top right corner. For H&E and Masson trichrome staining, Magnification, 200×; Bar = 100 μm. For TEM images, Bar = 200 nm. **b** Collagen fibrils diameters were measured according to TEM images. **c** Maximum load, Young’s Modulus, and strain at break of in vitro engineered cell-matrix complex were tested. Significant differences existed between two groups labeled with different letters. All error bars indicate standard deviation.

The engineered neo-tendon tissues were cultured under both static and dynamic conditions. As shown in Fig. 5a, after 4 weeks of in vitro culture, no obvious difference in gross appearance was found among all groups after static culture or dynamic culture with mechanical loading. However, further histological analysis suggested that relatively mature tissues were formed in the PLCL/COL+Fmod group, especially under dynamic culture condition. In the static conditions, although cells in all three groups were able to infiltrate into the core area of the scaffold and secrete ECM components, there was still no substantial progress in tissue maturation. TEM images showed relatively mature collagen fibrils in PLCL/COL and PLCL/COL+Fmod groups with more obvious periodic cross-striations and larger fibril diameters (Fig. 5a, b, *p* < 0.05).

Substantial changes in histology were initiated after 4 weeks of dynamic culture under mechanical loading in a bioreactor. The primitive tissue formed in the superficial layer of the PLCL/COL+Fmod scaffold with 3 to 4 layers of stacked cells. However, there was no obvious histologic structure in PLCL and PLCL/COL groups, indicating the important role of Fmod in inducing tenogenic differentiation of hASCs (Fig. 5a). In general, fibril diameters were significantly increased by dynamic traction of cell scaffold constructs except for the PLCL/COL+Fmod group, suggesting other limiting factors for collagen assembly in vitro (Fig. 5b).

### Enhanced mechanical properties of in vitro engineered constructs with COL1 and Fmod modification

As shown in Fig. 5c and Supplementary Fig. 4, the maximal load in PLCL, PLCL/COL, and PLCL/COL+Fmod groups were respectively 3.1 ± 0.8 N, 4.3 ± 0.6 N, 13.2 ± 0.9 N under static condition (*p* < 0.05), and respectively 5.6 ± 0.5 N, 7.6 ± 0.1 N, 18.9 ± 2.2 N under dynamic loading with significant differences among three groups (*p* < 0.05). Significance of difference in the maximal load could be observed between each group (*p* < 0.05), except for the difference between PLCL and PLCL/COL group under static condition. The maximal load in each group under dynamic stretching was significantly higher than that of the corresponding group under static condition (*p* < 0.05).

The Young’s modulus of PLCL, PLCL/COL, and PLCL/COL+Fmod groups were 17.1 ± 2.1 MPa, 51.6 ± 11.0 MPa, 81.9 ± 8.4 MPa, respectively, under static condition, and 2.3 ± 0.2 MPa, 5.1 ± 0.8 MPa, 22.8 ± 0.8 MPa, respectively, under dynamic loading among three groups (*p* < 0.05). Significant difference in Young’s modulus could be observed between each group (*p* < 0.05), except for the difference between PLCL and PLCL/COL group under dynamic stretching. However, the Young’s modulus in each group under static condition was significantly higher than that of the corresponding group under dynamic stretch (*p* < 0.05).

The strain at break of PLCL, PLCL/COL, and PLCL/COL+Fmod groups were 8.9 ± 1.0%, 7.9 ± 0.4%, 31.5 ± 1.1%, respectively, under static condition, and 45.6 ± 0.4%, 24.8 ± 1.4%, 37.5 ± 2.7%, respectively, under dynamic loading among three groups (*p* < 0.05). Significant difference in the strain at break was observed between each group (*p* < 0.05), except for the difference between PLCL and PLCL/COL group under static condition. The strain at break of each group under static condition was significantly lower than that of the corresponding group under dynamic stretch (*p* < 0.05). Taken together, the best mechanical performance of the engineered tendons was demonstrated in the PLCL/COL+Fmod group was proved in this assay.

### COL1 and Fmod accelerate tenogenic remodeling of in vivo implanted hASCs-yarns constructs

**Fig. 6:**
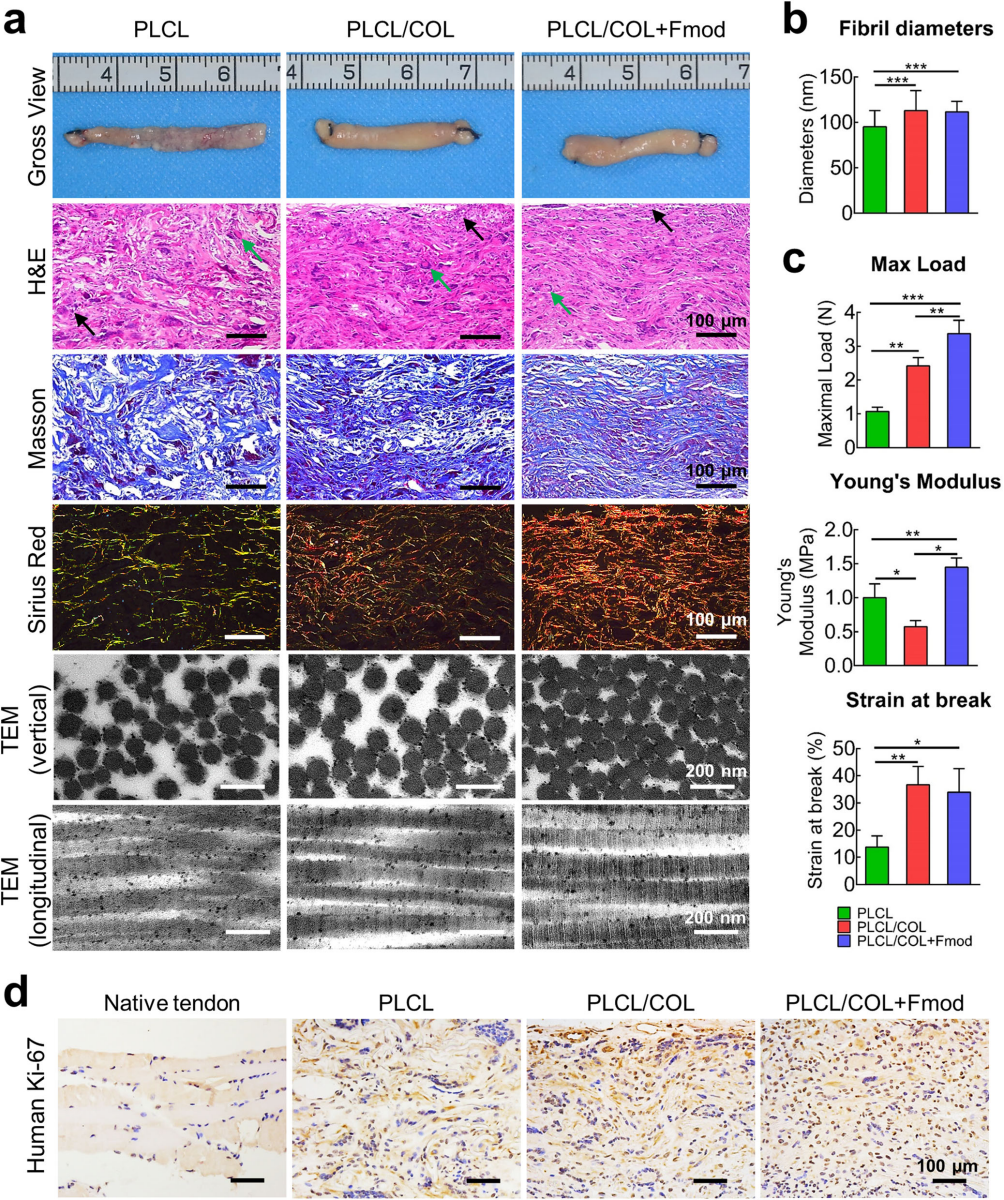
COL1 and Fmod dual-modified nanoyarn scaffold facilitated in vivo neo-tendon tissue formation. **a** Gross view, histological examination and ultrastructure of in vivo engineered tendon at the 6th week after implantation of cell-scaffold complex. The inflammatory cells and foreign body giant cells are indicated by solid arrows in black and green, respectively. For histochemical staining, Magnification, 200×; Bar = 100 μm. For TEM observation, Bar = 200 nm. **b** Collagen fibrils diameters were measured according to TEM images. **c** Maximum load, Young’s Modulus, and strain at break of in vivo engineered neo-tendon tissues at the 6th weeks post-implantation. *p < 0.05, **p < 0.01, ***p < 0.001. **d** Immunohistochemical staining of human Ki-67 revealed the highest viability of hASCs in PLCL/COL+Fmod group. Magnification, 200×; Bar = 100 μm. All error bars indicate standard deviation.

As a combination of ECM molecules with a stronger ability to induce tenogenic differentiation of hASCs, COL1 and Fmod also promoted the tissue maturation of hASCs-nanoyarns constructs upon in vivo implantation. As shown in Fig. 6a, after 6 weeks of in vivo implantation, a much thicker tendon tissue with higher tissue volume was observed grossly in both PLCL/COL and PLCL/COL + Fmod groups grossly, whereas the tissue was poorly developed in the PLCL group.

Histologically, parallel collagen fibers with a relatively organized tissue pattern were observed in both PLCL/COL and PLCL/COL+Fmod groups. In particular, elongated cells similar to those in native tendon were observed in the PLCL/COL+Fmod groups. In contrast, a random tissue pattern was observed in the PLCL group, where randomly distributed cells were observed. In addition, foreign body giant cells and inflammatory cells were observed along with undegraded material fragments, indicating adverse cell-material interaction and poor compatibility of the unmodified PLCL fibers.

Relatively, much denser collagen matrices were observed in H&E and Masson’s staining in the PLCL/COL groups compared to other two groups. Interestingly, a much higher level of tissue maturation was observed only in the PLCL/COL+Fmod group, as predominant type I collagen was found exclusively in this group (yellow-colored fibers in polarized photomicrographs), indicating the potent tenogenic effect of these two specific ECM molecules.

### COL1 and Fmod improved collagen fibril ultrastructure of neotendons in vivo

The TEM examination revealed the densest collagen fibrils in PLCL/COL+Fmod group, which was consistent with the histologic results (Fig. 6a). Although D-band periodicity was obviously observed in all groups, collagen fibril assembly of neotendons in vertical section was significantly improved in PLCL/COL group and PLCL/COL+Fmod group as revealed by fibril diameter (Fig. 6a, b, *p* < 0.05). Quantitatively, the average fibril diameters of neotendons in PLCL, PLCL/COL and PLCL/COL+Fmod groups were 95.2 ± 18.0 nm, 113.0 ± 21.9 nm, and 111.5 ± 11.6 nm, respectively with significant difference among three groups (Fig. 6c, *p* < 0.05), but no significant difference was found between collagen fibrils in PLCL/COL group and PLCL/COL+Fmod group. Unfortunately, the polarization of collagen fiber diameter distribution, which is usually considered as another ultrastructural feature of tendon^21^, was not found in all three groups.

### COL1 and Fmod enhanced biomechanical performance of neotendons in vivo

The maximal load, Young’s modulus and Strain at break were significantly increased in the in vivo formed tendons by COL1 and Fmod. Quantitatively, as shown in Fig. 6c, the maximal load of neotendons in PLCL, PLCL/COL and PLCL/COL+Fmod groups was 1.1 ± 0.1 N, 2.4 ± 0.3 N and 3.4 ± 0.4 N, respectively with significant difference among three groups (*p* < 0.05). The Young’s Modulus of neotendons in PLCL, PLCL/COL and PLCL/COL+Fmod groups was 1.0 ± 0.2 MPa, 0.6 ± 0.1 MPa and 1.5 ± 0.1 MPa, respectively with significant difference among three groups (*p* < 0.05). The Strain at break of neotendons in PLCL, PLCL/COL and PLCL/COL+Fmod groups was 13.7 ± 4.2%, 36.7 ± 6.7% and 33.9 ± 8.8%, respectively, with significant difference among three groups (*p* < 0.05). However, no significant difference in Strain at break was detected between PLCL/COL and PLCL/COL+Fmod groups.

### COL1 and Fmod facilitated hASCs survival in cell-scaffold complex

Further immunohistochemical examination of human Ki-67 showed the highest survival rate of hASCs in the PLCL/COL+Fmod group after 6 weeks of in vivo implantation (Fig. 6d). As shown by human Ki-67 immunohistochemical staining, a negative result was observed in the native Achilles tendon of nude mice, as the positive rate was less than 5%. However, the most abundant Ki-67-positive cells were observed in the PLCL/COL+Fmod group, while there were significantly fewer cells in the PLCL and PLCL/COL groups.

### COL1 and Fmod enhanced tenogenic-specific differentiation of hASCs on nanoyarn scaffolds

**Fig. 7:**
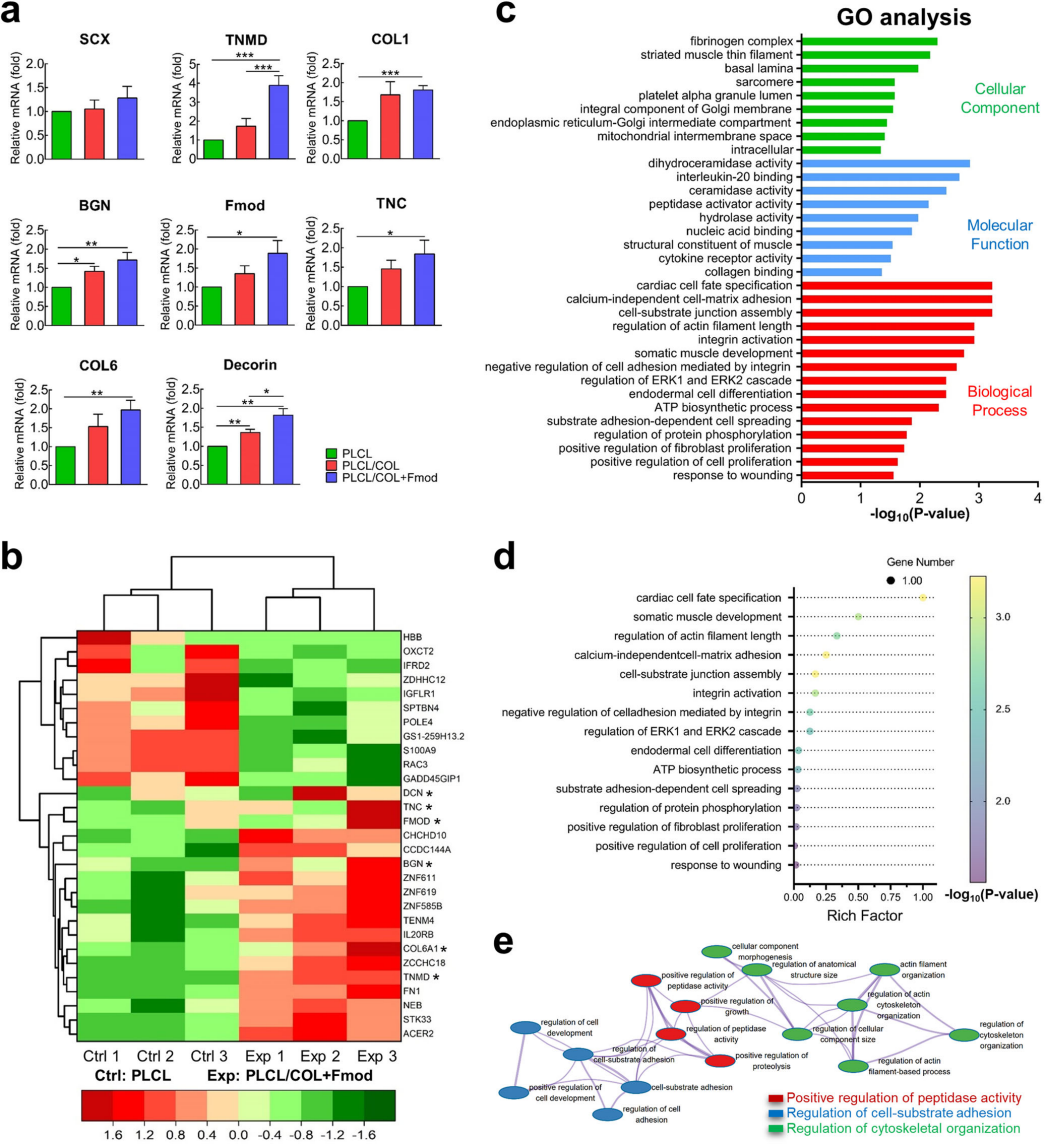
GO analysis revealed the transcriptomic alterations amid tenogenic differentiation process of hASCs. **a** hASCs that were seeded on PLCL/COL+Fmod scaffold expressed significantly higher levels of tenogenic genes. *p < 0.05, **p < 0.01, ***p < 0.001. **b** Differentially expressed mRNAs and representative tenogenic mRNAs were displayed in a clustered heat map. *p < 0.05 but FDR ≥ 0.05. **c** Representative GO terms enriched by differentially expressed mRNAs regarding Cellular Component, Molecular Function and Biological Process. **d** Bubble chart showed the degree of enrichment of 15 representative Biological Process terms. **e** All the Biological Process terms enriched by differentially expressed mRNAs were integrated into an interaction network as shown. All error bars indicate standard deviation.

After culturing on nanoyarn scaffolds for 7 days, the expression levels of tenogenic genes, including TNMD, COL1, COL6, Fmod, TNC, BGN, and DCN, were significantly increased on the dual-modified scaffold compared to those in the control group (Fig. 7a, *p* < 0.05). In contrast, modification of solo COL1 was only able to increase the expression levels of BGN and DCN (Fig. 7a, *p* < 0.05). In addition, there is no significant difference in the expression level of SCX among all groups, although an increasing trend was observed in the PLCL/COL+Fmod group (*p* > 0.05).

On the other hand, no obvious enhancement for differentiation trend towards other lineages was observed on dual-modified scaffold, such as chondrogenic (Supplementary Fig. 5a), osteogenic (Supplementary Fig. 5b) and adipogenic (Supplementary Fig. 5c) lineages. Only the expression levels of *aggrecan* and *ALPL* were higher in the PLCL/COL+Fmod group than in the PLCL group (*p* < 0.05). These results indicated the predominant tenogenic phenotype of hASCs on the PLCL/COL+Fmod scaffold.

### GO analysis of differentially expressed mRNAs indicated their functionality in tenogenic phenotype establishment

Considering the differentially expressed tenogenic genes between PLCL group and PLCL/COL+Fmod group, RNA-seq analysis was used to evaluate the whole mRNA profile of hASCs. As shown in Fig. 7b, a total of 23 differentially expressed mRNAs, including 11 downregulated and 12 upregulated mRNAs, were identified strictly by fold change and FDR value to avoid false positive results. These differentially expressed genes, together with several tenogenic genes already identified by qPCR (marked with * in Fig. 7b), were further categorized by a cluster heat map.

These identified differentially expressed mRNAs were then classified into biological process, molecular function, and cellular component for GO analysis. Representative GO terms are listed in Fig. 7c. Regarding biological process, COL1 and Fmod mainly led to differential enrichment in cell-ECM adhesion, cytoskeleton organization, cell fate decision, protein biosynthesis and cell proliferation (Fig. 7c, d), which also supports the results observed in Figs. 4, 5 and 6. In terms of molecular function, COL1 and Fmod mainly regulated protease activity and membrane receptor binding activity. For cellular components, COL1 and Fmod mainly induced changes in intracellular membrane components and cytoskeletal filaments.

The interaction network of all enriched Biological Process terms was then auto-generated after labeling with different titles such as positive regulation of peptidase activity, regulation of cell-substrate adhesion, and regulation of cytoskeletal organization (Fig. 7e), which primitively elucidated the different biological behaviors underlying the induction of the tenogenic phenotype.

### ceRNA regulation axis was constituted based on differentially expressed lncRNAs, miRNAs and mRNAs

**Fig. 8:**
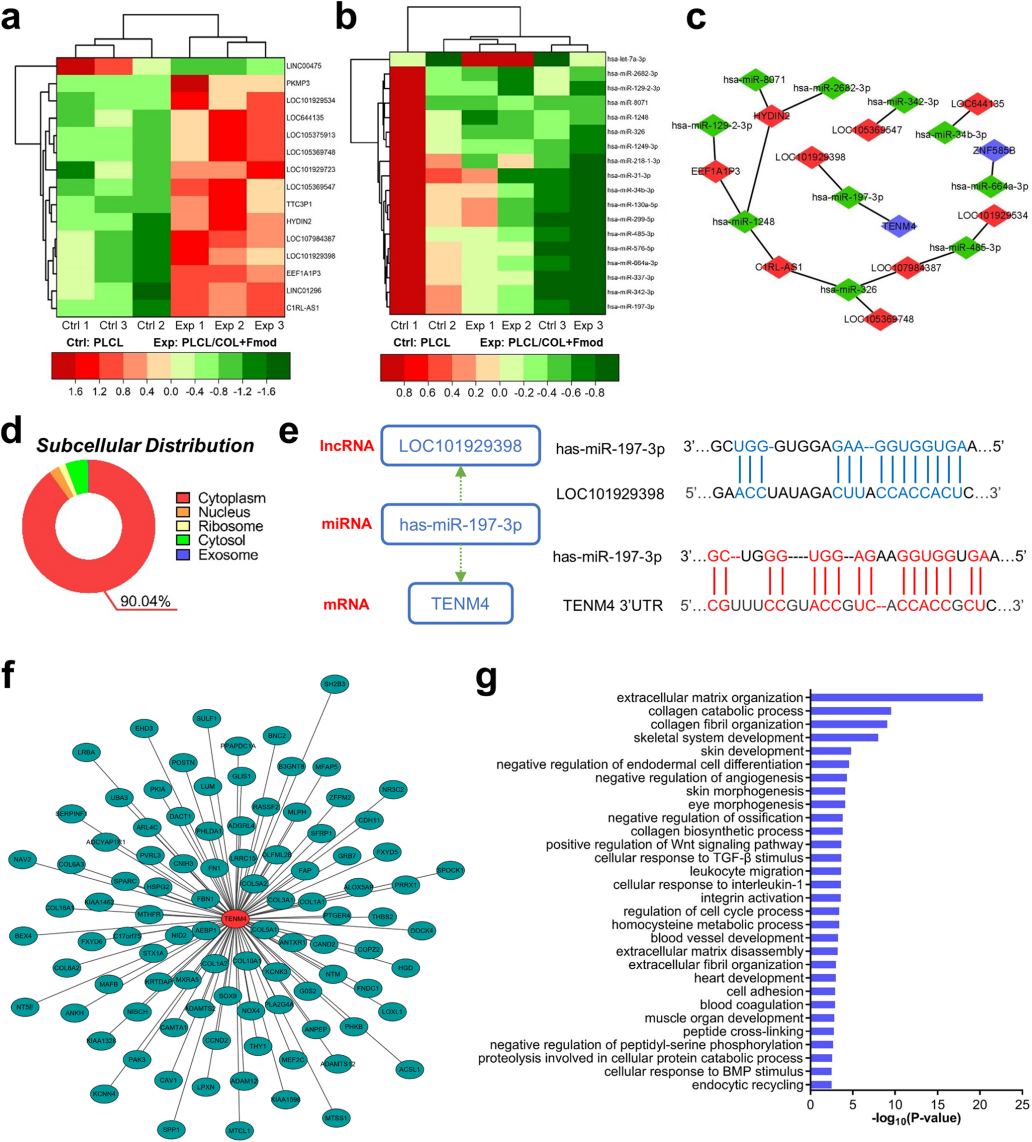
The ceRNA regulation axis was predicted to potentially affect the tissue/organ morphogenesis. **a** Differentially expressed lncRNAs were displayed in a clustered heat map. **b** Differentially expressed miRNAs were displayed in a clustered heat map. **c** The triple regulation network showed the interaction relationship among differentially expressed mRNAs, lncRNAs and miRNAs. Blue hub indicates mRNA. Red hub indicates lncRNA. Green hub indicates miRNA. **d** Subcellular distribution of *LOC101929398* was exhibited as a pie chart. **e** Possible binding sequences between *LOC101929398* and *has-miR-197-3p*, *TENM4* and *has-miR-197-3p* were predicted. 3′UTR, 3′-untranslated region. **f** Genes co-expressed with TENM4 were identified. Longer distance from central hub indicates lower correlation score. **g** Top 30 of GO Biological Process terms enriched by these co-expressed genes were illustrated in a histogram.

The same filtering criteria (log2(fold change) >0.585 or < −0.585; FDR < 0.05) were also used to identify differentially expressed lncRNAs and miRNAs. In total, 15 differentially expressed lncRNAs were selected, of which 14 were upregulated and 1 was downregulated (Fig. 8a). Similarly, 18 differentially expressed miRNAs, including 1 upregulated and 17 downregulated, were identified as shown in Fig. 8b. Potential target miRNAs of these differentially expressed lncRNAs and mRNAs were predicted and double-checked using miRanda and RNAhybrid tools. Finally, a total of 9 lncRNAs, 10 miRNAs and 2 mRNAs were found to be involved in the lncRNA-miRNA-mRNA triple regulatory network and then visualized by Cytoscape software (Fig. 8c). Among them, the *LOC101929398-has-miR-197-3p-TENM4* axis formed a complete ceRNA regulatory axis. The subcellular location of this regulatory axis, where *LOC101929398* may act as a ceRNA to stabilize *TENM4*, was predicted to be the cytoplasm (90.04% probability) according to the base sequence of *LOC101929398*, suggesting that *LOC101929398* is likely to exert regulatory effect by sponging has-miR-197-3p in the cytoplasm ((Fig. 8d). Furthermore, the target RNA binding sequences between has-miR-197-3p and *LOC101929398* or *has-miR-197-3p* and the 3′-untranslated region of *TENM4* were predicted as shown in Fig. 8e.

### Genes co-expressed with TENM4 were closely correlated with organ development

To further elucidate the biological process that might be affected by *TENM4*, genes correlated with *TENM4* were identified through co-expression analysis. As revealed in Fig. 8f, a total of 97 correlated genes were identified and arranged by correlation score. The longer distance indicated less relevance, and vice versa. These genes were principally enriched in 178 GO biological processes regarding tissue development and morphogenesis, extracellular matrix organization and cell proliferation. The top 30 representative GO biological process terms were unraveled in Fig. 8g. Briefly, most of these terms support trauma healing as well as regeneration of various types of mesenchymal tissues from the cellular level to the organ level, especially of the skeletal-muscle motor system, while facilitating the reduction of calcification formation of soft tissue.

**Fig. 9:**
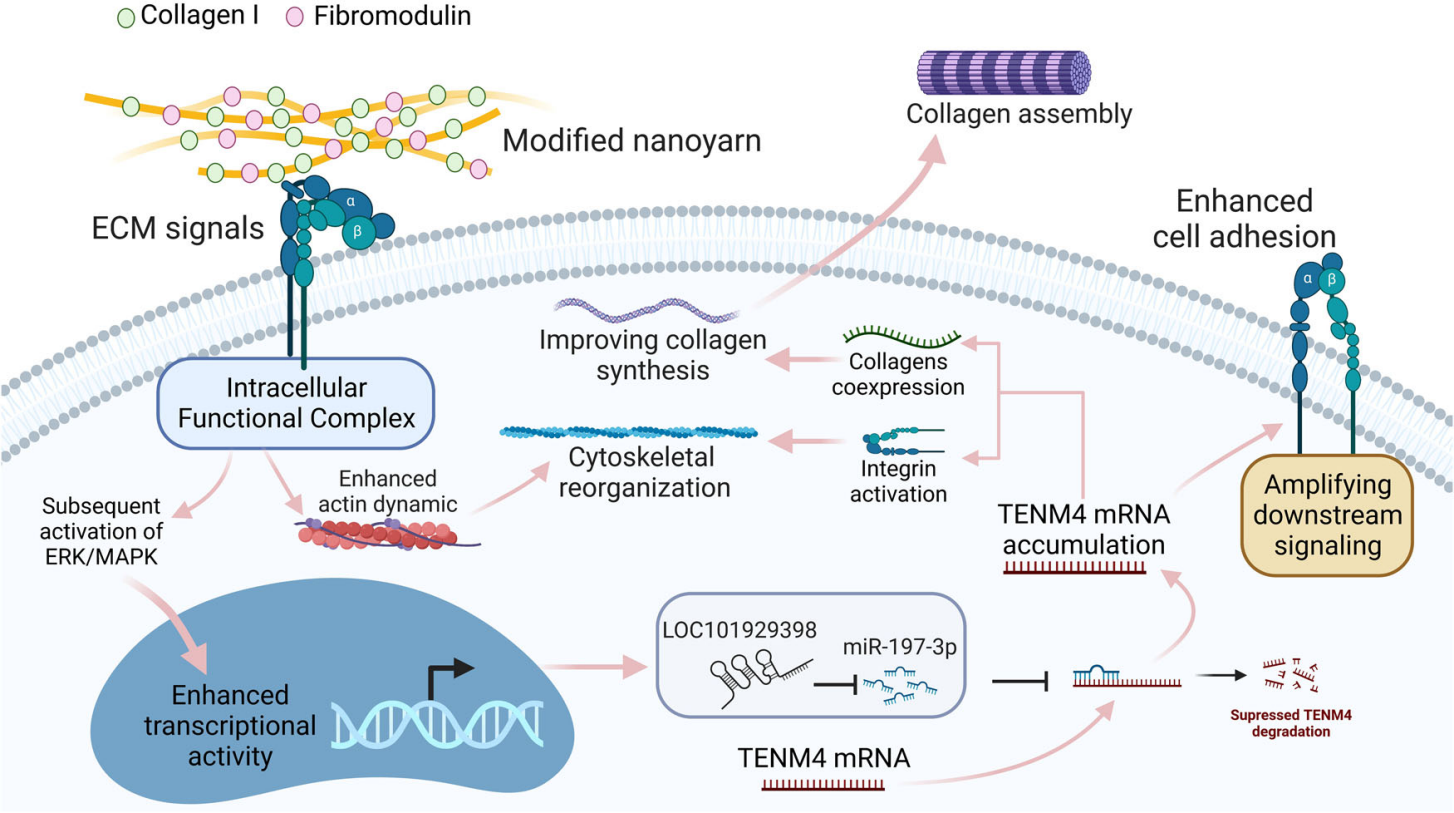
Schematic illustration summarized the proposed cellular biological mechanism by which COL-Fmod modified nanoyarn scaffold may induce tenogenic differentiation of hASCs through *LOC101929398*/*has-miR-197-3p*/*TENM4* ceRNA network. The ECM signals provided by COL1 and Fmod are considered as the vital niche signals that increases expression levels of *LOC101929398* and *TENM4*, and then trigger ceRNA regulation mechanism as shown. Intracellular accumulation of *TENM4* mRNA was predicted to serve as the core event during the establishment of tenogenic phenotype in hASCs. By co-expression of collagens and integrin activation, accumulated *TENM4* mRNA enhances collagen secretion, cytoskeletal reorganization and cell adhesion, thus confers tenogenic traits on hASCs.

Based on the above findings, the possible mechanism by which COL-FOMD dually modified nanoyarns induced the tenogenic phenotype of hASCs was deduced and illustrated in Fig. 9.

## Discussion

Engineering tendon grafts by seeding stem cells on bioinductive materials have gained tremendous interest as the alternatives to autografts transplantation due to less donor sites morbidity^22^. Amid this process, stem cells were required to stably adhere onto scaffold and thus could be integrated into a cell-scaffold complex^23^. Although the synthetic fibers were usually reported as superior in mechanical property and structural flexibility, obvious shortcomings such as limited cell adhesion and in vivo aseptic inflammation still need to be solved^24^. Unlike the synthetic materials, tendon-derived natural ECM that is much more biocompatible provides unique biochemical cues through stable cell-matrix interaction^25^. To restore the microenvironment that mimics the native tendon ECM, it has been considered to reinforce synthetic fibers by introducing natural tendon ECM components at the surface interface^26^. However, the cumbersome preparation process and the complicated chemical composition of tendon ECM extracts have hindered further mechanism exploration, owing to the undetermined key molecules that constitute the niche environments for tenogenic differentiation. Actually, more efficient and precise scaffold designs could be realized by modifying critical inductive molecules of tendon ECM other than total ECM extracts.

Unfortunately, no study has yet provided an efficient solution in the context of tissue engineering about ECM molecules that can serve as equivalents of vital niche factors. Attributing to frequent reports regarding the supportive effect of COL1 and SLRPs family on tendon development and remodeling^14,27^, simplified combinations of these representative molecules were designed to validate their tenogenic inductive effect. To mimic the mechanism of cell-ECM interaction, a widely adopted substrate-coating model was used for screening the combinations of molecules with teno-potent capacity^28^. The primary screening study showed that both COL1 and Fmod as a combination were the most suitable set of molecules for in vitro tenogenic induction of hASCs in the conventional 2D culture system and also exhibited teno-potent activity on the substrate with lower stiffness and aligned topography as shown in Fig. 2. Based on the results of preliminary screening experiments, this combination of molecules was used for subsequent modification of polymer nanoyarn scaffolds to facilitate further improvement of scaffold induction properties based on a biomimetic tendon microenvironment structure. Further study also confirmed that the COL1 and Fmod dual-modified nanoyarn scaffolds played a similar role in promoting tenogenic differentiation of hASCs in a three-dimensional environment as revealed in Fig. 7a. Although hASCs in the PLCL/COL group expressed slightly higher levels of tenogenic genes compared to the control group, no significant differences were detected in most cases, which showed a pattern similar to the COL group *versus* the control group in the 2D culture model.

It has been reported that Fmod collaborates with collagen in normal tendon tissue to maintain tendon tissue homeostasis and biomechanical function^14^. In present study, negative feedback regulation of COL1 synthesis probably mediated via several transmembrane receptors was canceled by Fmod^29,30^, leading to consistent extracellular collagen deposition. Fmod also accelerated cell adhesion and spreading along nanoyarns in vitro and collagen fibrils assembly in vivo like previously reported^31,32^. These biological processes were considered significantly important to tendon grafts formation. On the other hand, in addition to the direct phenotypic induction, the supporting effect of Fmod with COL on the seed cells in the constructs allowed the subcutaneous formation of tendon-like tissue in nude mice. As shown in Fig. 6d, human Ki-67-labeled hASCs remained dominant in the Fmod-COL dual-modified nanoyarn scaffolds and the highest positive rate indicated vigorous proliferative viability in the dual-modification group. However, hASCs were significantly reduced in the single COL modified group and the control group.

Further, the present study confirmed that exogenous tension stimulation enhanced the ability of Fmod with COL to induce tendon regeneration at the three-dimensional level. As observed in Fig. 5, matrix production of hASCs in static culture system was obviously inadequate for preliminary tissue formation, implicating a stagnant tissue formation process. By contrast, the ability of hASCs to secrete collagen could be enhanced by dynamic stretch stimulation, representing a fundamental mechanism whereby the grafts can continue to maintain the load-bearing structures^33^. Thicker layers of collagen deposits can be observed on COL1-Fmod modified scaffolds under dynamic loading stress, which initially exhibited a hierarchically stratified pattern. Moreover, the elongated hASCs resided within the gaps between collagen layers, also indicates that COL1-Fmod modification facilitated the formation of neotendons under dynamic stimulation in vitro.

Even though periodic loading stress weakened the stiffness of the nanoyarn material in general^34^, the grafts in PLCL/COL+Fmod group still showed comparatively stronger stiffness and limited increment in maximal strain under dynamic stimulation. It is speculated that a large amount of collagen deposited under dynamic stimulation may fill in the cracks and fine necks of the electrospun fibers, which in turn strengthens the Young’s modulus of the constructs through forming more hydrogen bonds^35^. Similar results were also observed during nanoyarn electrospinning (Fig. 3h). These phenomena once again confirmed the cornerstone role of mechanical stimulation amid maturation of engineered neo-tendons as previous reported^7,18^.

More profound RNA-seq and GO analysis suggested that phenotype shift caused by COL1 and Fmod might be initiated through stronger cell-substrate adhesion, then promoted by multiple peptidases and eventually realized by reorganization of cytoskeleton. Our previous studies have demonstrated that physically enforced alignment of cytoskeleton directed the predominant tenogenic phenotype of dermal fibroblasts^36^. However, stable cell adhesion also cannot be ignored according to above findings in current study. The tenogenesis of stem cells induced by tendon ECM was realized by integrin/TGF-β crosstalk as previously reported^9^, which was also revealed as GO terms in current study (i.e., integrin activation & regulation of ERK1/ERK2 cascade). Given this, the two ECM molecules were likely to promote tenogenic transformation of hASCs mediated by intensifying cell-scaffold binding and subsequent activation of canonical TGF-β signaling.

With the establishment of further ceRNA regulatory axis, the regulatory target of these functional changes at the transcriptional level, *TENM4*, was predicted. *TENM4* can be co-expressed with a variety of cell differentiation and fate decision genes (as shown in Figs. 8 and 9), which suggests that the crosstalk between hASCs and COL1-Fmod couple may affect cell phenotype by regulating the expression level of *TENM4*. Studies on mouse embryos have found that *TENM4* mutations will cause mesoderm dysplasia during early embryo development^37^, but its function for tendon development still need further elucidation. It is currently known that *TENM4* is a transmembrane protein that plays an important role in the development of nerves, muscles but inhibit the formation of cartilage tissue^38–42^, which was in accordance with current research.

Among them, *TENM4* mediates nervous system development by promoting cytoskeletal reorganization and axon formation^41^, a process similar to the establishment of tenogenic phenotype in hASCs after unidirectional cell extension. Here, based on the results of this study, the authors presumed that COL1 and Fmod may upregulate *TENM4* expression levels through a ceRNA mechanism and then accelerate the cytoskeletal dynamics. Meanwhile, the *TENM4* may also be co-expressed with other genes involved in the tissue development and cellular response to TGF-β1, thus accelerating the remodeling of engineered tendon tissue at the histological level. Based on the results of the bioinformatics predictions, a possible mechanism theory of how *TENM4* mRNA accumulation induces tenogenic differentiation in hASCs was speculated according to our co-expression analysis (Figs. 8g and 9). As there is no original literature reported on tendon regeneration, future investigations can focus on the functionality of *TENM4* in native tendon development and engineered tendon graft formation.

There are certainly imperfections in this research. For example, the effective ECM molecular combinations were designed based on literature evidence rather than on high-throughput screening experiments. Investigators can draw on more bioinformatics and artificial intelligence tools to calculate better scaffold modification schemes in subsequent work. Meanwhile, although a single Fmod-modified nanoyarn scaffold may also theoretically facilitate tendon differentiation, it is actually difficult to immobilize Fmod directly on aliphatic polyester scaffolds via the NHS/EDC reaction. The results of this study also need to be further validated in large animal models as well as in situ regeneration models of tendons.

In conclusion, the current study identifies COL1 and Fmod in combination for the first time as the teno-potent ECM molecules required for the tenogenic differentiation of hASCs and preliminarily demonstrates the functional enrichment of hASCs induced by both COL1 and Fmod in a 3D tissue construct model. In this proof-of-concept ECM modification model, the expression level of *TENM4* was predicted to serve as a core regulatory target through which COL and Fmod induce hASCs into a tenogenic phenotype. Future research will focus on functionally verification of *TENM4* and its ceRNA regulatory axis at different levels of tissue architecture, and elucidating the regulatory mechanism of how COL1 and Fmod trigger the crosstalk between integrin and TGF-β signaling pathway.

## Methods

### Topographical surface and scaffold materials

The PDMS membranes with parallel microgrooves or smooth surface were kindly provided by Dr. James Wang of the University of Pittsburgh. Those parallel grooves on PDMS membranes were 10-μm in width and 3-μm in depth and thus could enforce the aligned morphology of cells. PLCL (LA:CL = 75:25, Mw = 300,000) was provided by Daigang Biomaterial Co. (Jinan, China). Solid COL1 was extracted from rat tail tendons as previously described^43^. Recombinant human Fmod was purchased from Sino Biological (Beijing, China). The 1,1,1,3,3,3-hexafluoro-2-propanol (HFIP), which served as a solvent of PLCL and COL1, was purchased from Shanghai Aladdin Biochemical Technology Co., Ltd. 2-(N-morpholino) ethanesulfonic acid (MES) was provided by Solarbio (Beijing, China). NHS and EDC were purchased from Thermo Fisher Scientific (MA, USA).

### Isolation, maintenance, and characterization of hASCs

Isolation of hASCs was performed according to the previously published protocol^44^. All following procedures of handling human fat tissue was approved by the Ethic Committee of Shanghai Ninth People’s Hospital affiliated to Shanghai Jiao Tong University School of Medicine (HKDL[2018]234). Briefly, human adipose tissues were harvested from abdomen of five patients who received plastic surgery and were informed with written consents for tissue donation. Tissue samples were washed in chloramphenicol and then cleaned with PBS. After been minced, tissue granules were digested with 0.1% collagenase NB4 (SERVA Electrophoresis GmbH, Heidelberg, Germany) which was dissolved in Dulbecco’s modified eagle medium (DMEM, Hyclone, UT, USA) containing 10% fetal bovine serum (FBS, Gibco, NY, USA). After digestion at 37 °C for 1 h with vigorous shaking, cells were centrifuged at 500 g for 5 min then washed in PBS. Cells were subsequently resuspended in DMEM containing 10% FBS and 1% Antibiotic-Antimycotic solution (Gibco; Thermo Fisher Scientific) before being seeded on petri dish. Cells were maintained with DMEM containing 10% FBS and 1% Antibiotic-Antimycotic solution at 37°C in a humidified atmosphere containing 95% air and 5% CO_2_. Once cells reached 90% confluency, these primary cells were detached with 0.25% trypsin-EDTA (Gibco), centrifuged and passaged at a density of 10^4^/cm^2^. Cells of passage 2 to passage 4 were employed in this study.

Cellular phenotype was verified by incubating cells of passage 2 with various phycoerythrin labeled pre-diluted monoclonal antibodies such as anti-human CD45 (BD Pharmingen, 555483), anti-human CD31 (BD Pharmingen, 555446), anti-human HLA-DR (BD Pharmingen, 555812), anti-human CD90 (BD Pharmingen, 555596), anti-human CD105 (BD Pharmingen, 560839), anti-human CD13 (BD Pharmingen, 560998), anti-human CD73 (BD Pharmingen, 550257), and anti-human CD44 (BD Pharmingen, 561858). Cells were collected and gently washed after an incubation at 37 °C for 30 min. Afterward, the resuspended cells were analyzed in a flow cytometer (Thermo Fisher Scientific) as previously described^45^.

### Screening of teno-potent ECM molecules on hASCs-seeded 2D substrates

The tenogenic induction capacity of COL1, BGN, Fmod and DCN was assessed by examining expression levels of tenogenic markers in hASCs. Passage 2 hASCs were seeded on petri dishes coated with following ECM molecule groups as listed in Table 1. The COL1 gel was provided by Xinyou Biotechnology (Hangzhou, China). The recombinant Fmod, BGN, DCN as well as FN proteins were purchased from Sino Biological (Beijing, China). The initial cell density was 2000 cells/cm^2^. DMEM containing 10% FBS and 1% Antibiotic-Antimycotic solution was used for cell culture for 7 days, followed by detection of expression levels of tenogenic genes.

**Table 1:** The ECM molecules used for substrate coating.

Group Name	Coated Molecules
Control	Null
COL1	10 μg/cm^2^ of collagen I gel
BGN	4 ng/cm^2^ of biglycan
Fmod	4 ng/cm^2^ of fibromodulin
DCN	4 ng/cm^2^ of decorin
FN	4 ng/cm^2^ of fibronectin
Mixture	4 ng/cm^2^ of biglycan, fibromodulin, decorin and fibronectin
COL1 + BGN	10 μg/cm^2^ of COL1 gel plus 4 ng/cm^2^ of biglycan
COL1 + FN	10 μg/cm^2^ of COL1 gel plus 4 ng/cm^2^ of fibromodulin
COL1 + DCN	10 μg/cm^2^ of COL1 gel plus 4 ng/cm^2^ of decorin
COL1 + Mixture	10 μg/cm^2^ of COL1 gel plus 4 ng/cm^2^ of biglycan, fibromodulin, decorin and fibronectin

To further test the induction effect of ECM molecules on hASCs with elongated morphology, cells were subsequently seeded on COL1 or Fmod coated PDMS membrane with smooth or parallelly microgrooved surface according to protocols described in previous study^36^. Briefly, hASCs were seeded onto membranes at a density of 2000 cells/cm^2^ and then cultured with DMEM containing 10% FBS and 1% Antibiotic-Antimycotic for 7 days. In the control group, PDMS membranes were coated with 4 ng/cm^2^ of FN. Tenogenic differentiation of hASCs was examined at the 7th day similarly. Three biological replicates were involved in this assay.

### Scaffold fabrication and modification

Fabrication of PLCL and PLCL/COL nanoyarn scaffolds were conducted in an electrospinning system with dynamic liquid as previously described^10^. Briefly, spontaneous water vortex occurred in the basin when water drained through a small hole (8 mm in diameter) on the bottom of the basin. Meanwhile, water in the tank was pumped back to the basin to keep a stable water level. PLCL was dissolved in HFIP to yield a single working solution (8 w/v%), or dissolved in HFIP with COL to yield a blending working solution (8 w/v%) for subsequent fabrication of PLCL or PLCL/COL nanoyarn, respectively. The two kinds of working solution were delivered into a spinneret at a constant rate of 1.0 mL/h and electrospun under a high voltage of 15 kV. The gap between spinneret and water vortex was set as 15 cm. Electrospun nanofibers were generated after HFIP evaporated and deposited on the water; then, bundles of nanoyarn formed after the nanofibers were twisted in the water vortex and collected by a mandrel rotating at 60 rpm to generate comparatively aligned nanoyarns. The nanoyarn scaffold was frozen at –80 °C for 2 h and subsequently dried overnight in a vacuum oven. The final nanoyarn mesh was about 1.50 mm thick and stored at room temperature before use.

To prepare PLCL/COL+Fmod nanoyarn, Fmod was immobilized on PLCL/COL scaffold via EDC/NHS crosslinking reaction. Crosslinking reaction was performed under an acid condition (pH = 5.5) to minimize the hydrolysis of EDC. The working solution for crosslinking reaction was prepared by dissolving Fmod, EDC and NHS in MES buffer sequentially and the final concentrations of Fmod, EDC, and NHS were 0.5 μg/mL, 30 mM, and 8 mM, respectively. Filter-sterilization of the working solution was performed through a 0.22-μm syringe filter unit (Millipore, MA, USA) before use. To realize the crosslinking reaction, 1 cm^2^ of nanoyarn film was immersed in 100 μL working solution at room temperature for 2 h and then washed in phosphate buffered saline (PBS) for at least 3 times. A remnant of free reactant was removed by soaking films in PBS overnight. The modified scaffold was aired before used in following assays.

### Characterization of nanoyarn scaffolds

Surface morphology of nanoyarns was observed by scanning electron microscopy (SEM, BAL-TEC, Philips, Eindhoven, The Netherlands) after being sputter-coated with gold. Fifty single fibers which constituted yarns were randomly selected and measured for their diameters and angles between two adjacent yarns via ImageJ software (version 1.8.0; National Institutes of Health). The hydrophilicity of nanoyarns was assessed by recording water spreading process on the surface of nanoyarns. The continuous images revealed a drop of water (0.3 mL) spreading on the surface of the samples. Water contact angles at 30 s of the spreading process were measured for three technical replicates in directions perpendicular to the orientation of the nanoyarns.

To verify the chemical composition of the nanoyarn scaffolds, Fourier transform infrared (FTIR) spectra were performed to examine the scaffolds and the corresponding inclusive monomers, and were recorded continuously from 4000 cm^−1^ to 500 cm^−1^ with an attenuated total reflection accessory (ATR-FTIR, Thermo Nicolet, Waltham, MA, USA). X-ray photoelectron spectroscopy (XPS, Thermo Electron Corporation, Escalab 250, Germany) was also used to identify binding energy peaks of C_1s_, N_1s_, O_1s_, and S_2p_ at 282, 398, 528, and 168 eV, respectively. For PLCL/COL+Fmod nanoyarn, different concentrations (50 ng/mL, 500 ng/mL, 5000 ng/mL) of Fmod in the working solution were set to elucidate the dose-dependent effect of Fmod concentration on crosslinking efficacy.

The mechanical properties of nanoyarns were measured using a biomechanical analyzer (Instron 4411, Canton, MA, USA), where modified and rectangular scaffolds (1.5 cm long, 0.5 cm wide) were subjected to constant stretching along the yarn orientation at a rate of 5 mm/min until break. Maximum load, Young’s modulus, and strain at break were calculated from the automatically generated stress-strain curves. Four technical replicates were used in this test.

### Cell morphology and proliferation on nanoyarns

Cells were seeded on nanoyarns with DMEM plus 10% FBS and 1% antibiotic-antimycotic solution at a density of 5000 cells/cm^2^. The cytoskeleton was stained with rhodamine-conjugated phalloidin (Yeasen Biotech, Shanghai, China) and then counterstained with 4,6-diamidino-2-phenylindole (DAPI, Life Technology, CA, USA) to visualize the nuclei on the 1st, 3rd, 5th and 7th day of cell culture. The cell aspect ratios of fifty cells were measured using ImageJ software and their frequency distribution was calculated. Similarly, cells were incubated with Cell Counting Kit-8 (CCK-8, Dojindo, Japan) on the 1st, 3rd, 5th, and 7th day to detect their proliferation rates as previously reported^46^. Four biological replicates were included in this assay.

### Live/dead staining

Cells were seeded on nanoyarns at a density of 5000 cells/cm^2^ and cultured with DMEM plus 10% FBS and 1% antibiotic-antimycotic solution. After 7 days of culture, cells were incubated for 30 min at 37 °C with 2 μM Calcein-AM (AAT Bioquest, CA, USA) and 4.5 μM propidium iodide (Yeasen Biotech) which were dissolved in phenol red-free medium. Residual dye was removed before cells were observed by fluorescence microscopy.

### Quantitation of intracellular collagen production

Cells were seeded and cultured on nanoyarns as described above. At the 7th day of culture, cells were detached then collected in the Eppendorf tubes. The cytomembrane was dissociated by 1% Triton X-100 (Sigma-Aldrich; Merck KGaA) for 30 min. Intracellular collagen production was quantified using a hydroxyproline quantification kit (Jian Cheng Bioengineering Institute, Nanjing, China). Five biological replicates were included in this assay.

### SEM of cell-scaffold construct

As previously reported^7^, samples were fixed in 2.5% glutaraldehyde at 4 °C overnight and then washed in PBS, followed by dehydration in increasing concentrations of ethanol (25%, 50%, 75%, 95%, and 100% for 15 min, respectively). Afterward, samples were soaked in tertiary butanol for 10 min, which was repeated for 3 times. The remaining liquid was thoroughly removed by lyophilization prior to sputtering gold onto the samples. SEM photographs were then captured.

### Static engineering of tendon

As described previously^47^, disinfected nanoyarn scaffolds (4.5 cm long, 1.5 cm wide and 0.15 cm thick) were attached to custom-made stainless-steel clamps under the aseptic conditions. To avoid floating beyond the surface of the culture medium, the two ends of the scaffolds were naturally stretched. The stretched scaffolds were then transferred to 15-cm Petri dishes. After being seeded with hASCs at a density of 1.2 × 10^6^ cells/scaffold, the constructs were cultured with DMEM plus 10% FBS and 1% antibiotic-antimycotic solution for 4 weeks. The frequency of medium replacement was once a week. Cell-scaffold constructs were harvested for various experiments.

### Tendon engineering under dynamic mechanical loading

Cell-scaffold constructs were prepared as described in previous section. After 7 days of in vitro culture for cell adhesion, the cell-scaffold constructs were unloaded from stainless-steel rings and anchored in a culture chamber. Uniaxial mechanical stretching was employed in this study. One end of the construct was fixed to the stator while the other end of the construct was connected to shifting bar, which enabled periodical elongation and rebounding of the constructs with dynamic mechanical loading. Up to 4 constructs were allowed simultaneously during in vitro culture with dynamic circulation of 1 L of culture medium consisting of DMEM plus 10% FBS and 1% antibiotic-antimycotic solution at a flow rate of 60 ml/h in the closed loop. The constructs were stretched at 4% elongation along the yarns’ orientation at a frequency of 0.5 Hz. Loading was performed three times daily (9:00 to 10:00, 14:00 to 15:00, and 20:00 to 21:00) consecutively for 4 weeks. Samples were then harvested for various experiments.

### Animal model and in vivo tendon engineering

Cell-scaffold constructs for in vivo tendon engineering were prepared by seeding hASCs on nanoyarns at a density of 8 × 10^5^ cells/cm^3^. The cell-scaffold constructs were first cultured in vitro for 7 days before in vivo implantation. A total of 27 male nude mice (~18 g, 6 weeks old) were purchased from Shanghai Slaccas Experimental Animal Co., Lit.. Three groups were set in the present study: PLCL group in which hASCs-PLCL constructs were implanted subcutaneously; PLCL/COL group in which hASCs-PLCL/COL constructs were implanted subcutaneously; PLCL/COL+Fmod group in which hASCs-PLCL/COL+Fmod constructs were implanted subcutaneously. After randomly numbered, equal numbers of nude mice were randomly assigned to the three groups described above (*n* = 9 in each group). The surgical procedures and animal management were approved by the Animal Care and Experiment Committee of Shanghai Ninth People’s Hospital affiliated to Shanghai Jiao Tong University School of Medicine. First, animals were anesthetized by inhalation of 4.5% isoflurane (0.8 L/min in air) and then maintained by inhalation of 1.5% isoflurane (0.6 L/min in air). For subcutaneous embedding of the cell scaffold constructs according to the previous protocol^48^, subcutaneous fistulae in dorsal aspect were made bilaterally between the anterior limb and the ipsilateral posterior limb on both sides under the aseptic conditions. The cell-scaffold constructs were rolled up in the direction perpendicular to yarns orientation. Rod-shape grafts were inserted into the subcutaneous fistulae, then both ends of the constructs were sutured to the muscle fascia with 5-0 non-absorbable sutures. Animals were kept under specific pathogen free condition after recovery from anesthesia and had access to approach their food and water. Animals were sacrificed at 6 weeks post implantation. Newly formed tissues were harvested for various studies.

### Histological analysis

Harvested samples were fixed in 4% paraformaldehyde, dehydrated in ethanol and embedded in paraffin. Tissue samples were sectioned at 5-μm thickness and stained with H&E staining kit (Beyotime Biotechnology), Sirius red staining kit (Solarbio), and Masson’s trichrome staining kit (Jian Cheng Bioengineering Institute, Nanjing, China) for histological analysis according to the product instructions.

### Immunohistochemical staining

To assess the viability of hASCs in the subcutaneously implanted constructs, human Ki-67 positive cells were visualized by immunohistochemical staining. Tissue sections (5 μm thick) of in vivo engineered tendon samples were prepared as aforementioned above, and nude mouse Achilles tendon samples were used as a negative control. Briefly, after inactivation of endogenous peroxidase, antigen retrieval, and blotting with 10% goat serum, the tissue sections were incubated with rabbit-anti-human Ki-67 antibody (Abcam, ab92742, diluted at 1:200 in PBS) at 4 °C overnight, followed by visualization with the GTVisionTM III detection system/Mo&Rb (Dako, K5007). Images were captured through a light microscope.

### Transmission electron microscopy (TEM)

Tissue samples were fixed in 2.5% glutaraldehyde at 4 °C for at least 24 h. To visualize the striation and diameter of collagen fibrils, samples were processed according to standard procedures for TEM examination (Quanta 200 FEI, Eindhoven, The Netherlands). Images were captured by KS400 Image analysis software (version 3.0, Zeiss). The average diameters of 50 collagen fibrils, which were randomly selected from three different samples (*n* = 150) were measured via ImageJ and three individual biological samples were included in this assay.

### Biomechanical analysis of engineered tendons

The in vitro and in vivo engineered tissues were anchored to the clamps of a biomechanical analyzer (Instron 4411, Canton) and stretched at 10 mm/min along the nanoyarn orientation as previously reported^7^. Stress-strain curves were constantly recorded until complete rupture of tested tissue, followed by automatic generation of Maximal load (max load in Newtons [N]), Young’s modulus (MPa), and Strain at break (%). At least three individual biological specimens were involved in this assay.

### Tenogenic induction of hASCs on nanoyarn scaffolds

Cells were seeded on nanoyarns (untreated, COL1 modified and COL1-Fmod dual-modified) at a density of 10,000 cells/cm^2^ and cultured with DMEM plus 10% FBS and 1% antibiotic-antimycotic solution for 7 days. The expression levels of genes related to tenogenic and other mesenchymal lineages were detected by real-time quantitative polymerase chain reaction (qPCR). This assay included three biological replicates.

### RNA isolation and real-time qPCR

Total cellular RNA was extracted by lysing cells in TRIzol^®^ reagent (Thermo Fisher Scientific) and then quantified via spectrophotometer (Molecular Devices, CA, USA). Complementary DNA, which served as the templet for qPCR, was synthesized using the EZ-press reverse transcriptase kit (EZBioscience, MN, USA) with 1 μg of RNA samples when their A_260_/A_280_ values were between 1.8 and 2.0. The synthesis reaction was performed at 42°C for 15 min.

Fold changes in gene expression levels were calculated by qPCR analysis. qPCR was conducted with Power SYBR Green PCR Master Mix (2×) (EZBioscience) in a real-time thermal cycler (Q6, Applied Biosystems, CA, USA). Thermocycling procedures were set as recommended in the instruction manuals: 95°C for 5 min followed by subsequently 40 cycles (95 °C for 10 s then 60 °C for 30 s). The human housekeeping gene *GAPDH* was used as an internal control and each assay was performed in triplicate. All experiments were repeated on at least three different samples. The base sequences, annealing temperatures, and production sizes of human primers for real-time qPCR analysis are listed in Supplementary Table 1.

### RNA-seq

RNA-seq was used to analyze the transcriptional differences between hASCs cultured on PLCL scaffold (control group) and hASCs cultured on PLCL/COL+Fmod scaffold (experimental group). After depletion of ribosomal RNA, the total RNA with its integrity number above 8.0 was used to construct RNA-seq libraries by VAHTS Total RNA-seq (H/M/R) Library Prep Kit for Illumina (NEB, USA). For miRNA sequencing, the small RNA cDNA libraries were prepared using the TruSeq Small RNA Sample Prep Kit (Illumina, USA). Whole transcriptome sequencing was performed on the Hiseq™ XTEN Sequencer (Illumina, San Diego, CA, USA) with the assistance of NovelBio Corp. Laboratory (Shanghai, China). After filtering and mapping the clean reads to the human genome database (human genome version: GRCH38.p7, NCBI) and miRbase (version 22, University of Manchester), the differentially expressed mRNAs, lncRNAs and miRNAs were generated by *EB-Sequencing algorithms* from the raw data. Adjusted *p*-values were calculated using the *Benjamini–Hochberg’s approach* to control the false discovery rate (FDR). In addition, these differentially expressed RNAs were selected according to the following criteria: (1) log_2_(Fold Change) > 0.585 or < −0.585; (2) FDR < 0.05, and then visualized as a cluster heatmap. Three biological replicates were included in this assay.

### Gene set enrichment analysis

Differentially expressed RNAs were mapped to GO terms (http://www.geneontology.org/), and the network of these matched GO term clusters was analyzed using the online tool Matescape (http://metascape.org) as previously reported^49^. The network images involved in this article were designed by Cytoscape (Version 3.8.2, NIH).

### Prediction of lncRNA-miRNA-mRNA interactions, lncRNA location, RNA binding area and mRNA co-expression profile

In this study, the RNA regulation network was also constructed to understand the potential epigenetic regulation mechanism by the special ECM molecules. To construct the competing endogenous RNA (ceRNA) regulation network, miRanda v3.3a (http://www.microrna.org/microrna/home.do) and RNAhybrid 2.1 (https://bibiserv.cebitec.uni-bielefeld.de/rnahybrid/) were applied as the tools to predict target mRNAs and target lncRNAs for specific miRNA. The candidate mRNAs and lncRNAs detected by two tools simultaneously were considered qualified to reduce the false positive results. The subcellular location of lncRNA was predicted by lncLocator tool (http://www.csbio.sjtu.edu.cn/bioinf/lncLocator/), which was powered by Shanghai Jiao Tong University^50^. The RNA binding sequences of miRNA to mRNA 3′UTR and miRNA to lncRNA were predicted by RNA22 tool (version 2, Thomas Jefferson Computational Medicine Center, USA)^51^. Genes likely to be co-expressed with mRNA of ceRNA network were predicted by Coexpedia tool as previously described^52^.

### Statistical analysis

All assays were repeated on at least 3 different samples and data are presented as mean ± standard deviation (SD). Significant differences among multiple groups were judged by one-way ANOVA followed by Least Significant Difference post-hoc test. The *P*-value was calculated using software SPSS (version 22.0, SPSS Inc., IL, USA). Differences were considered statistically significant if the *p*-value was less than 0.05.

### Reporting summary

Further information on research design is available in the Nature Research Reporting Summary linked to this article.

### Supplementary information

SUPPLEMENTAL MATERIALS

Reporting Summary

## Data Availability

The data discussed in this publication have been deposited in NCBI’s Gene Expression Omnibus and are accessible through GEO Series accession number GSE246270. All data supporting the conclusions of this study are either provided in this published paper (and its Supplementary Information files) or available from the authors upon reasonable request.
