# Supplementary material for: Type I collagen and fibromodulin enhance the tenogenic phenotype of hASCs and their potential for tendon regeneration
Source: NPJ Regen Med. 2023 Dec 14;8:67. doi: 10.1038/s41536-023-00341-z (PMC10719373; doi:10.1038/s41536-023-00341-z)
Supplement: Supplementary file 1 — SUPPLEMENTAL MATERIALS [file 41536_2023_341_MOESM1_ESM.pdf]

Supplementary Table 1. Human primers used in quantitative PCR analysis.

Gene	Primer sequence (5'-3')	Annealing temperature(°C)	Product size (bp)
GAPDH	Sense: TCACCATCTTCCAGGAGCG	60	572
	Antisense: CTGCTTCACCACCTTCTTGA		
Scleraxis	Sense: CGTTGCCCAGGTGCGAGATGTAG	60	144
	Antisense: CGAGCGAGACCGCACCAACA		
Tenomodulin	Sense: TTGAAGACCCACGAAGTAGA	60	170
	Antisense: ATGACATGGAGCACACTTTC		
Collagen I	Sense: GGCGGCCAGGGCTCCGACCC	60	347
	Antisense: AATTCCTGGTCTGGGGCACC		
Collagen VI	Sense: CCCTGAGGCTTAACTTGCTG	60	388
	Antisense: CCAGGATTCCCTTTCTCTCC		
Fibromodulin	Sense: ATTGGTGGTTCCACTACCTCC	60	87
	Antisense: GGTAAGGCTCGTAGGTCTCATA		
Decorin	Sense: AATAACCGAAATCAAAGATGGAGAC	60	351
	Antisense: TCAGCAATGCGGATGTAGGAG		
Tenascin-C	Sense: TGACAGAAGTGACGGAAGAG	60	194
	Antisense: GATGGCAAATACACGGATAA		
Biglycan	Sense: CAGTGGCTTTGAACCTGGAG	60	93
	Antisense: GGGAGGTCTTTGGGGATGC		
SOX-9	Sense: AGCCGAAAGCGGAGCTCGAAACT	60	215
	Antisense: GCACTTAGGAAGGCGCGGGGT		
Collagen II	Sense: CTTGGGCACCTCGGGCTCCTTTAG	60	510
	Antisense: TCCCCGGCACTCCTGGCACTGAT		
Aggrecan	Sense: AGTATCATCAGTCCCAGAATCTAGCA	60	132
	Antisense: AATGCAGAGGTGGTTTCACTCA		
ALPL	Sense: AAACCGAGATACAAGCACTCCCAC	60	140
	Antisense: TCCGTCACGTTGTTCCTGTTCA		

OCN	Sense: ATGAGAGCCCTCACACTCCTCG	60	255
	Antisense: GTCAGCCAACTCGTCACAGTCC		
RUNX2	Sense: ACAGTAGATGGACCTCGGGA	60	113
	Antisense: ATACTGGGATGAGGAATGCG		
aP-2	Sense: AGGGCGAAGTCTAAAAATGGAG	60	114
	Antisense: TAGTGATGTGAGCAGGGTAACG		
Adiponectin	Sense: TATCCCCAACATGCCCATTCG	60	114
	Antisense: TAGGCAAAGTAGTACAGCCCA		
PPAR-γ	Sense: AGCCTCATGAAGAGCCTTCCA	60	110
	Antisense: ACCCTTGCATCCTTCACAAGC		
C/EBPα	Sense: AAGAAGTCGGTGGACAAGAACAG	60	70
	Antisense: TGCGCACCGCGATGT		

Supplementary Figure 1

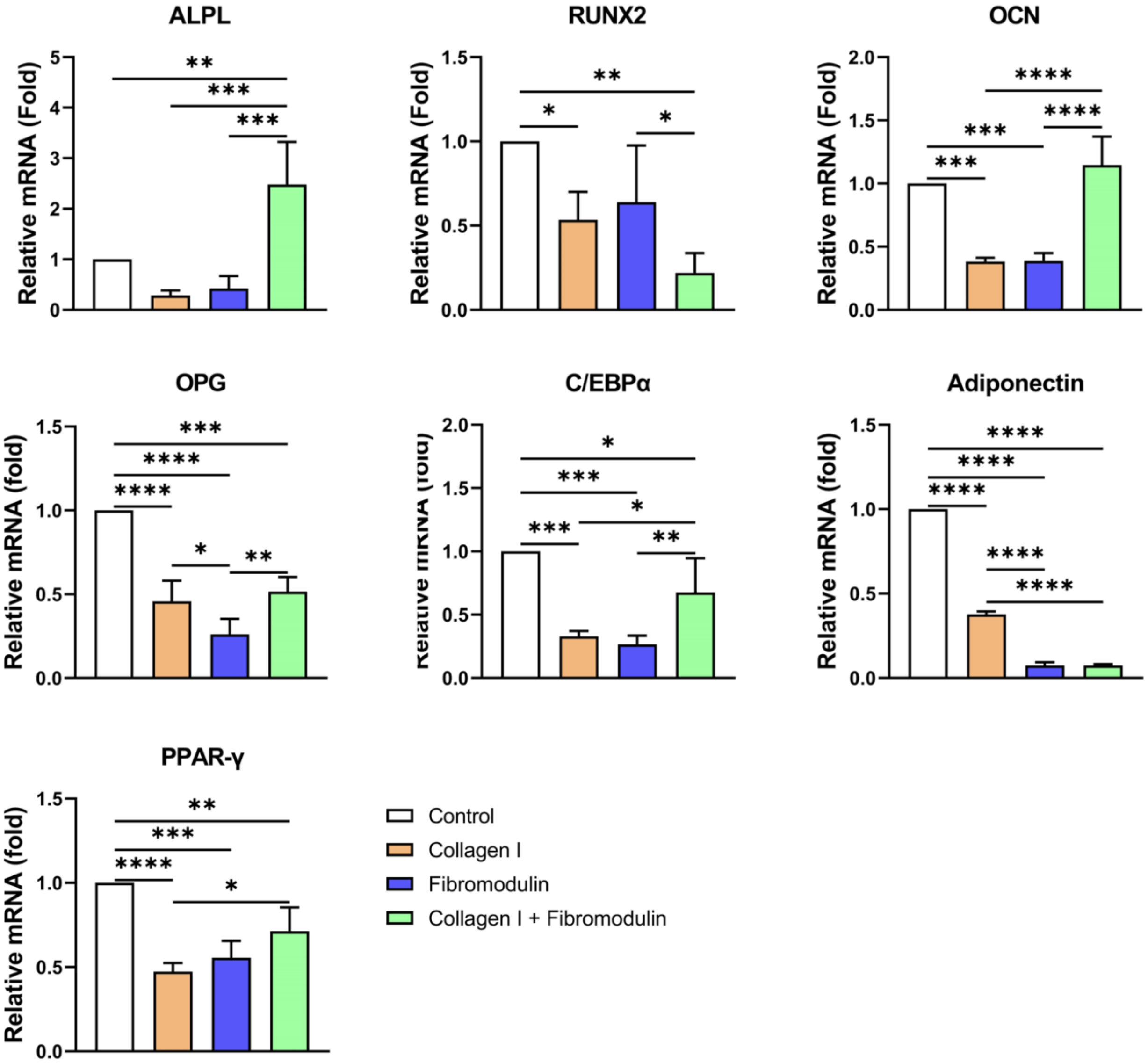

Supplementary Fig. 1 Type I collagen and fibromodulin did not significantly increase the osteogenic potential of hASCs in the substrate-coated model, while decreasing the pre-existing adipogenic differentiation capacity. Only the expression level of alkaline phosphatase was upregulated on the type I collagen and fibromodulin coated substrate. $*p < 0.05$, $**p < 0.01$, $***p < 0.001$, $****\ p < 0.0001$.

Supplementary Figure 2

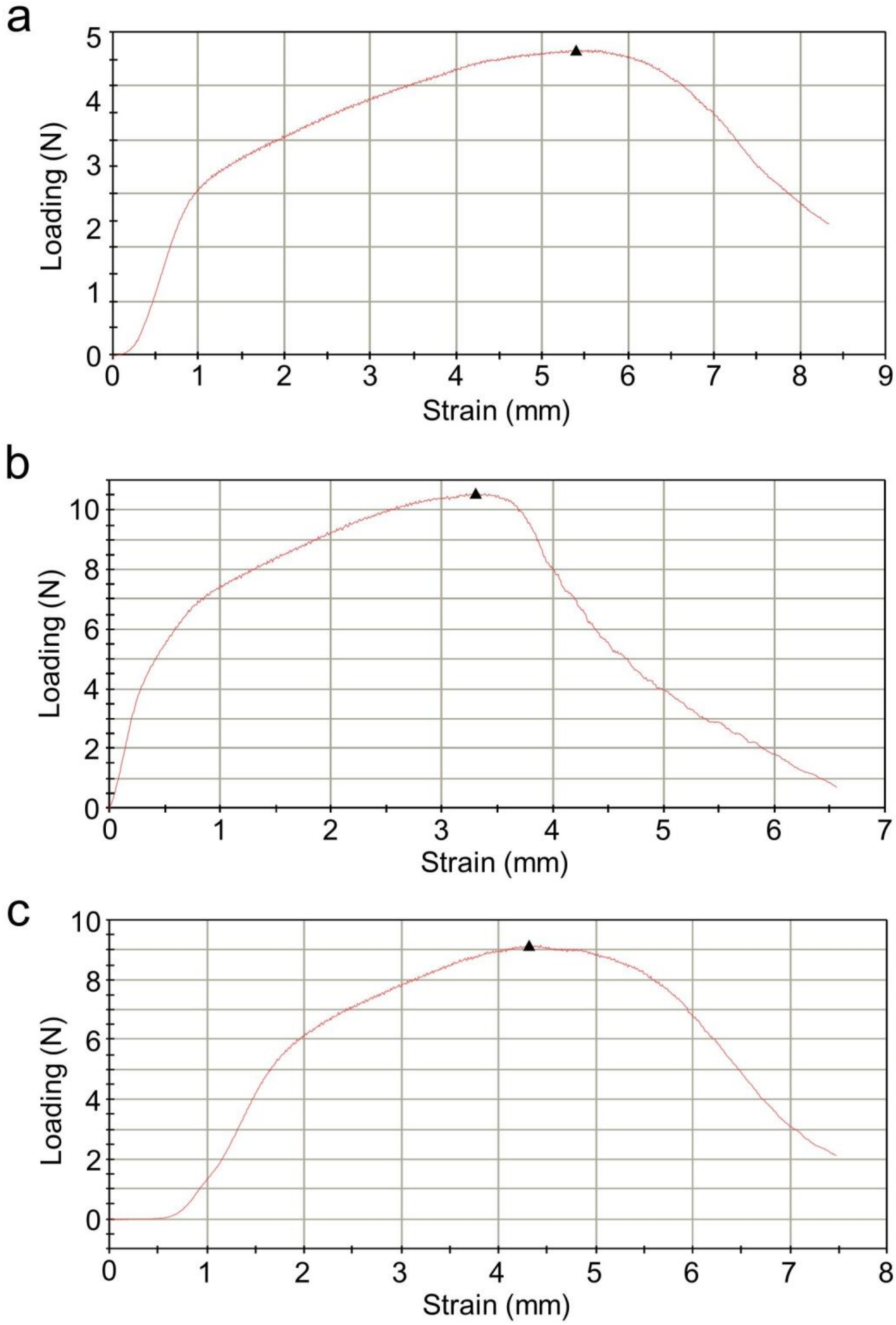

Supplementary Fig. 2 The representative stress-strain curves of nanoyarns including PLCL (a), PLCL/COL (b) and PLCL/COL+Fmod (c), respectively.

Supplementary Figure 3

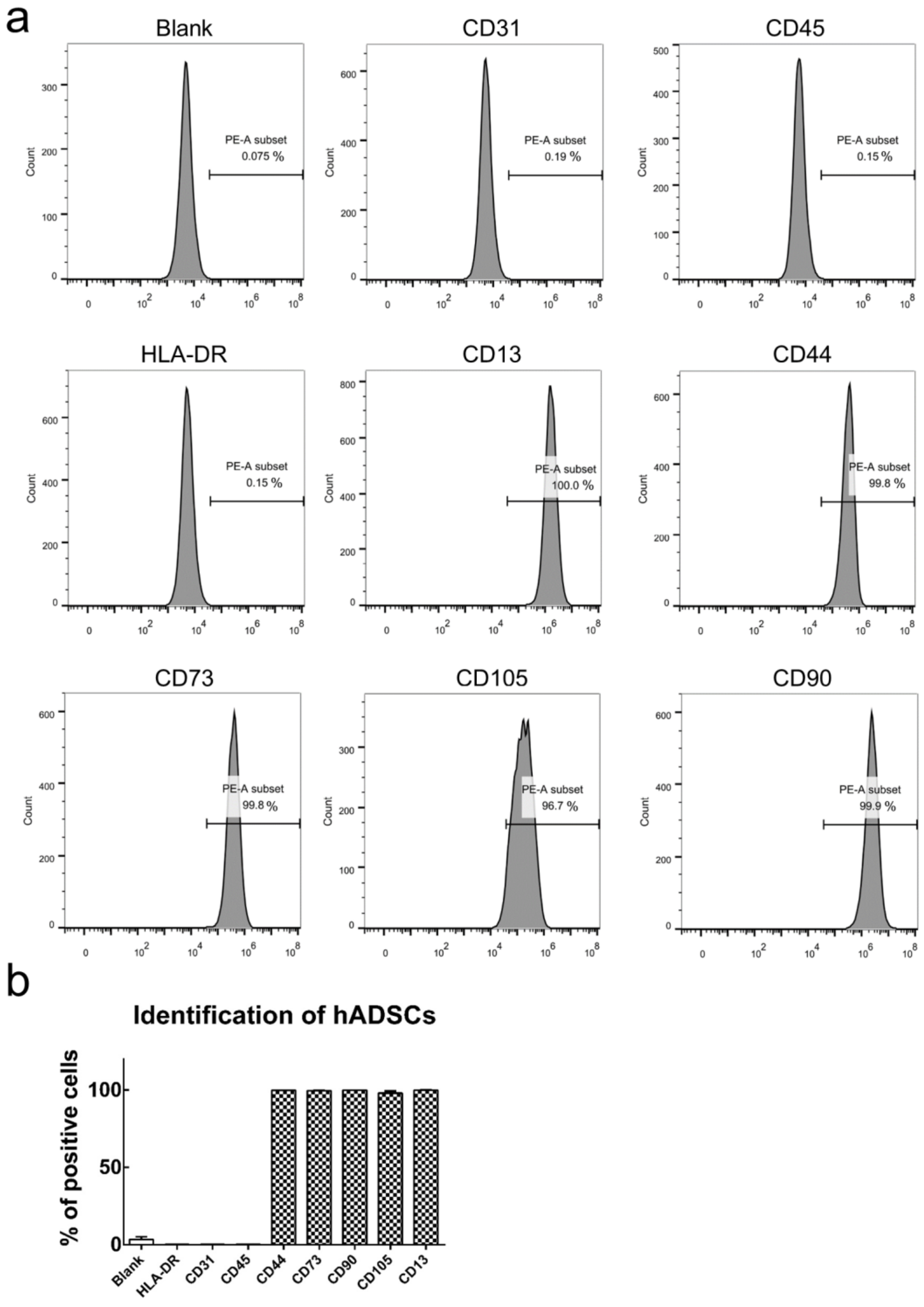

Supplementary Fig. 3 Identification of human adipose derived stem cells. (a) cytometry analysis revealed that cells expressed positive surface makers such as CD13, CD44, CD73, CD105 and CD90 but did not express CD31, CD45 and HLA-DR. (b) Positive rates for various surface markers were illustrated as a histogram. Three biological replicates were involved in this assay

Supplementary Figure 4

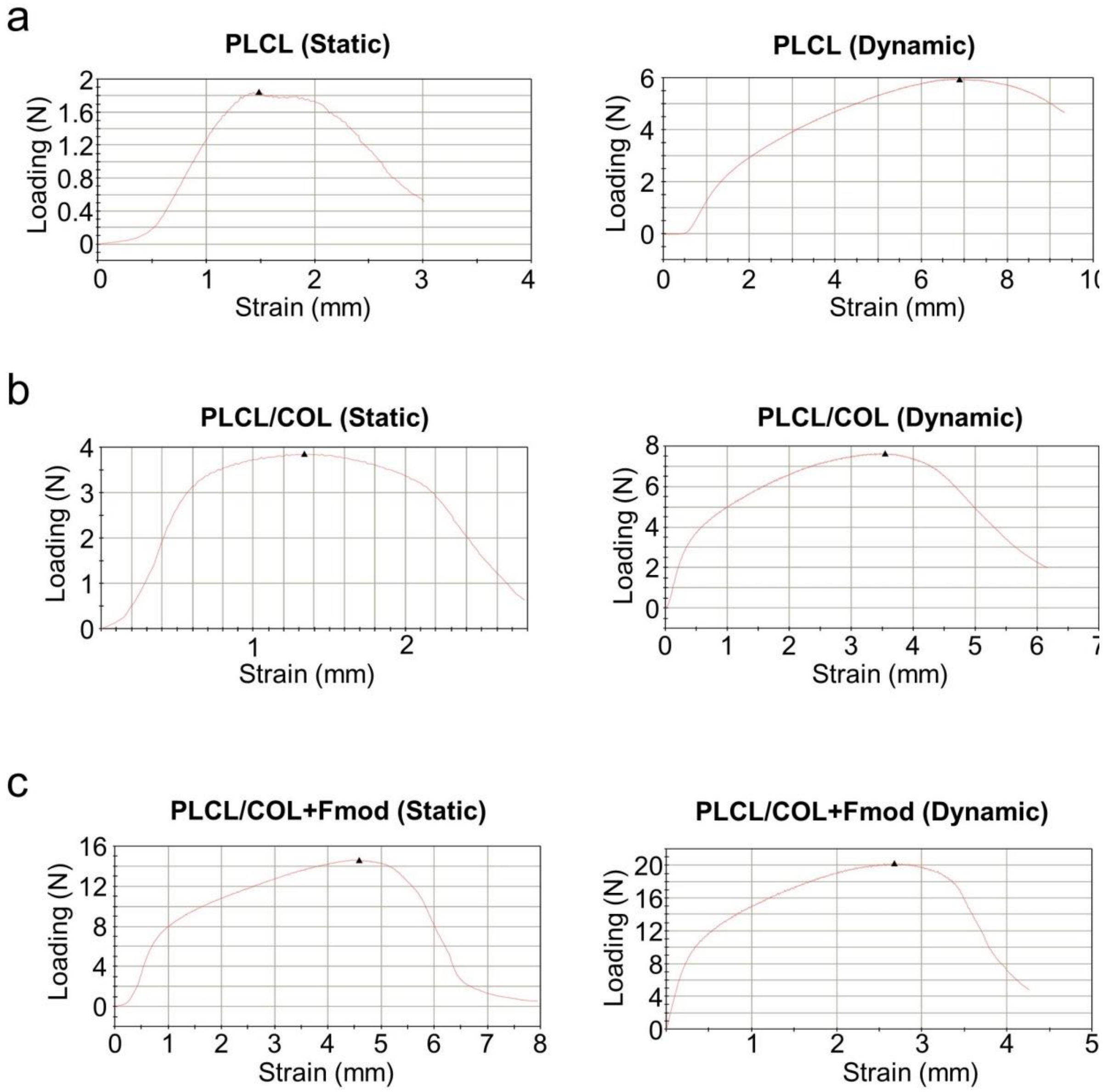

Supplementary Fig. 4 The representative stress-strain curves of *in-vitro* engineered tendon constructs in PLCL (a), PLCL/COL (b) and PLCL/COL+Fmod (c) groups, respectively

Supplementary Figure 5

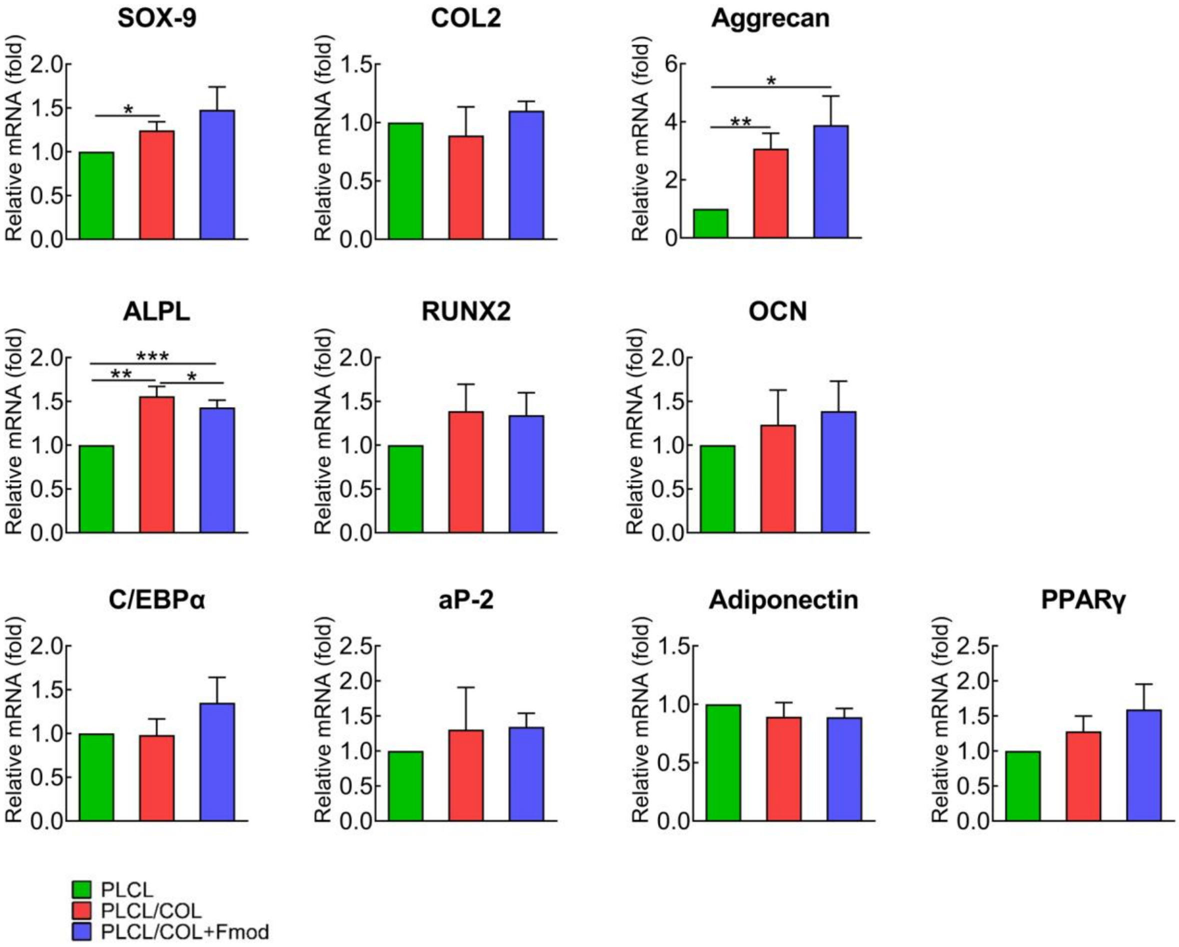

Supplementary Fig. 5 Expression levels of genes regrading multilineage differentiation on nanoyarns were detected at day 7. qPCR analysis revealed the alterations in expression levels of chondrogenic (a), osteogenic (b) and adipogenic (c) genes. Three biological replicates were involved in this assay. *$p < 0.05$, *$p < 0.01$, ***$p < 0.001$
