# Supplementary material for: Type I collagen and fibromodulin enhance the tenogenic phenotype of hASCs and their potential for tendon regeneration
Source: NPJ Regen Med. 2023 Dec 14;8:67. doi: 10.1038/s41536-023-00341-z (PMC10719373; doi:10.1038/s41536-023-00341-z)
Supplement: Supplementary file 2 — Reporting Summary [file 41536_2023_341_MOESM2_ESM.pdf]

nature portfolio

Corresponding author(s): Wei Liu

Last updated by author(s): Aug 18, 2023

Reporting Summary

Nature Portfolio wishes to improve the reproducibility of the work that we publish. This form provides structure for consistency and transparency in reporting. For further information on Nature Portfolio policies, see our Editorial Policies and the Editorial Policy Checklist.

Statistics

For all statistical analyses, confirm that the following items are present in the figure legend, table legend, main text, or Methods section.

n/a	Confirmed	
☐	☒	The exact sample size (*n*) for each experimental group/condition, given as a discrete number and unit of measurement
☐	☒	A statement on whether measurements were taken from distinct samples or whether the same sample was measured repeatedly
☐	☒	The statistical test(s) used AND whether they are one- or two-sided<br>*Only common tests should be described solely by name; describe more complex techniques in the Methods section.*
☐	☒	A description of all covariates tested
☐	☒	A description of any assumptions or corrections, such as tests of normality and adjustment for multiple comparisons
☐	☒	A full description of the statistical parameters including central tendency (e.g. means) or other basic estimates (e.g. regression coefficient) AND variation (e.g. standard deviation) or associated estimates of uncertainty (e.g. confidence intervals)
☐	☒	For null hypothesis testing, the test statistic (e.g. F, t, r) with confidence intervals, effect sizes, degrees of freedom and P value noted<br>*Give P values as exact values whenever suitable.*
☐	☒	For Bayesian analysis, information on the choice of priors and Markov chain Monte Carlo settings
☐	☒	For hierarchical and complex designs, identification of the appropriate level for tests and full reporting of outcomes
☐	☒	Estimates of effect sizes (e.g. Cohen's *d*, Pearson's *r*), indicating how they were calculated

Our web collection on statistics for biologists contains articles on many of the points above.

Software and code

Policy information about availability of computer code

Data collection	The related algorithms have been described in the Materials&Methods section.
Data analysis	The related algorithms have been described in the Materials&Methods section.

For manuscripts utilizing custom algorithms or software that are central to the research but not yet described in published literature, software must be made available to editors and reviewers. We strongly encourage code deposition in a community repository (e.g. GitHub). See the Nature Portfolio guidelines for submitting code & software for further information.

Data

Policy information about availability of data

All manuscripts must include a data availability statement. This statement should provide the following information, where applicable:

- Accession codes, unique identifiers, or web links for publicly available datasets
- A description of any restrictions on data availability
- For clinical datasets or third party data, please ensure that the statement adheres to our policy

All data supporting the conclusions of this study are either provided in this published paper (and its Supplementary Information files) or available from the authors upon reasonable request.

Research involving human participants, their data, or biological material

Policy information about studies with human participants or human data. See also policy information about sex, gender (identity/presentation), and sexual orientation and race, ethnicity and racism.

Reporting on sex and gender	This research does not included the content about sex-/gender-related results.
Reporting on race, ethnicity, or other socially relevant groupings	This research does not included the content related to the race, ethnicity, or other socially relevant groupings.
Population characteristics	This research does not involve the human research participants.
Recruitment	This research does not involve the human research participants.
Ethics oversight	Ethic Committee of Shanghai Ninth People's Hospital affiliated to Shanghai Jiao Tong University School of Medicine

Note that full information on the approval of the study protocol must also be provided in the manuscript.

Field-specific reporting

Please select the one below that is the best fit for your research. If you are not sure, read the appropriate sections before making your selection.

☒ Life sciences ☐ Behavioural & social sciences ☐ Ecological, evolutionary & environmental sciences

For a reference copy of the document with all sections, see nature.com/documents/nr-reporting-summary-flat.pdf

Life sciences study design

All studies must disclose on these points even when the disclosure is negative.

Sample size	Sample sizes were not calculated. We minimized the use of human tissues and experimental animals as much as possible, provided that the sample size was allocated according to the materials required for the experiment and the need to complete the statistical analysis.
Data exclusions	No data were excluded from the analyses.
Replication	All attempts at replication were successful.
Randomization	The animals were numbered and then allocated into different groups by generating computer-generated random numbers from 1 to 27.
Blinding	This research is not clinical trial so that there is no blinding.

Reporting for specific materials, systems and methods

We require information from authors about some types of materials, experimental systems and methods used in many studies. Here, indicate whether each material, system or method listed is relevant to your study. If you are not sure if a list item applies to your research, read the appropriate section before selecting a response.

Materials & experimental systems

n/a	Involved in the study	
☒	☐	Antibodies
☐	☒	Eukaryotic cell lines
☒	☐	Palaeontology and archaeology
☐	☒	Animals and other organisms
☒	☐	Clinical data
☒	☐	Dual use research of concern
☒	☐	Plants

Methods

n/a	Involved in the study	
☒	☐	ChIP-seq
☐	☒	Flow cytometry
☒	☐	MRI-based neuroimaging

Eukaryotic cell lines

Policy information about cell lines and Sex and Gender in Research

Cell line source(s)	The primary hASCs were isolated from five patients who received plastic surgery. These patients, including 2 males and 3

Cell line source(s)	females, donated their abdominal fat tissues.
Authentication	All of these primary cells were authenticated by flow cytometry analysis.
Mycoplasma contamination	All cell lines were tested negative for Mycoplasma contamination.
Commonly misidentified lines (See ICLAC register)	Not applicable

Animals and other research organisms

Policy information about studies involving animals; ARRIVE guidelines recommended for reporting animal research, and Sex and Gender in Research

Laboratory animals	Male nude mouse, 6 weeks old.
Wild animals	The study did not involve wild animal.
Reporting on sex	The results of this study is not sex-dependent and the male animal is commonly used when it comes to the nude mouse.
Field-collected samples	The study did not involve samples collected from the field.
Ethics oversight	The Animal Care and Experiment Committee of Shanghai Ninth People's Hospital affiliated to Shanghai Jiao Tong University School of Medicine

Note that full information on the approval of the study protocol must also be provided in the manuscript.

Flow Cytometry

Plots

Confirm that:

☒ The axis labels state the marker and fluorochrome used (e.g. CD4-FITC).

☒ The axis scales are clearly visible. Include numbers along axes only for bottom left plot of group (a 'group' is an analysis of identical markers).

☒ All plots are contour plots with outliers or pseudocolor plots.

☒ A numerical value for number of cells or percentage (with statistics) is provided.

Methodology

Sample preparation	After fixation in 70% alcohol, cells were incubated with PE-labeled antibodies and then analysed in a flow cylometer.
Instrument	CytoFLEX S, Beckman Coulter
Software	CytExpert for DxFLEX
Cell population abundance	There is no cell sorting procedure in this study.
Gating strategy	The gate control policy is based on a standardized policy provided by the equipment manufacturer. A negative control group was set up in this experiment to distinguish between negative and positive expressions.

☒ Tick this box to confirm that a figure exemplifying the gating strategy is provided in the Supplementary Information.
